# Red blood cell membrane-camouflaged nanoparticles loaded with AIEgen and Poly(I : C) for enhanced tumoral photodynamic-immunotherapy

**DOI:** 10.1093/nsr/nwab039

**Published:** 2021-03-03

**Authors:** Jun Dai, Meng Wu, Quan Wang, Siyang Ding, Xiaoqi Dong, Liru Xue, Qingqing Zhu, Jian Zhou, Fan Xia, Shixuan Wang, Yuning Hong

**Affiliations:** Department of Obstetrics and Gynecology, Tongji Hospital, Tongji Medical College, Huazhong University of Science and Technology, Wuhan 430032, China; Department of Obstetrics and Gynecology, Tongji Hospital, Tongji Medical College, Huazhong University of Science and Technology, Wuhan 430032, China; Engineering Research Center of Nano-Geomaterials of Ministry of Education, Faculty of Materials Science and Chemistry, China University of Geosciences, Wuhan 430074, China; Department of Chemistry and Physics, La Trobe Institute for Molecular Science, La Trobe University, Melbourne, Victoria 3086, Australia; Engineering Research Center of Nano-Geomaterials of Ministry of Education, Faculty of Materials Science and Chemistry, China University of Geosciences, Wuhan 430074, China; Department of Obstetrics and Gynecology, Tongji Hospital, Tongji Medical College, Huazhong University of Science and Technology, Wuhan 430032, China; Department of Obstetrics and Gynecology, Tongji Hospital, Tongji Medical College, Huazhong University of Science and Technology, Wuhan 430032, China; College of Material, Chemistry and Chemical Engineering, Hangzhou Normal University, Hangzhou 311121, China; Engineering Research Center of Nano-Geomaterials of Ministry of Education, Faculty of Materials Science and Chemistry, China University of Geosciences, Wuhan 430074, China; Department of Obstetrics and Gynecology, Tongji Hospital, Tongji Medical College, Huazhong University of Science and Technology, Wuhan 430032, China; Department of Chemistry and Physics, La Trobe Institute for Molecular Science, La Trobe University, Melbourne, Victoria 3086, Australia

**Keywords:** biomimetic drug delivery system, aggregation-induced emission, Poly(I : C), immunotherapy, photodynamic therapy

## Abstract

Red blood cell (RBC)-mimicking nanoparticles (NPs) offer a promising platform for drug delivery because of their prolonged circulation time, reduced immunogenicity and specific targeting ability. Herein, we report the design and preparation of RBC membrane-bound NPs (M@AP), for tumoral photodynamic-immunotherapy. The M@AP is formed by self-assembly of the positively charged aggregation-induced emission luminogen (AIEgen) (named P2-PPh3) and the negatively charged polyinosinic : polycytidylic acid (Poly(I : C)), followed by RBC membrane encapsulation. P2-PPh3 is an AIE-active conjugated polyelectrolyte with additional photosensitizing ability for photodynamic therapy (PDT), while Poly(I : C) serves as an immune-stimulant to stimulate both tumor and immune cells to activate immunity, and thus reduces tumor cell viability. When applied in tumor-bearing mice, the M@AP NPs are enriched in both the tumor region as a result of an enhanced permeability and retention (EPR) effect, and the spleen because of the homing effect of the RBC-mimicking shell. Upon light irradiation, P2-PPh3 promotes strong ROS generation in tumor cells, inducing the release of tumor antigens (TA). The anti-tumor immunity is further enhanced by the presence of Poly(I : C) in M@AP. Thus, this strategy combines the PDT properties of the AIE-active polyelectrolyte and immunotherapy properties of Poly(I : C) to achieve synergistic activation of the immune system for anti-tumor activity, providing a novel strategy for tumor treatment.

## Teaser

Immunotherapy has great potential, but the response of some tumors to immunotherapy is not high. Herein, we developed a biomimetic red cell membrane loaded with aggregation-induced emission luminogen and polyinosinic:polycytidylic acid nanoparticles to enhance the efficacy of tumor immunotherapy.

## INTRODUCTION

Immunotherapy is a type of anti-tumor treatment that harnesses the body's natural defense system to fight cancer. It has shown great clinical success against a wide variety of malignancies in recent years [1]. Current immunotherapy involves several different immune-based treatment methods. The most widely used effective immunotherapy so far is achieved with use of immune checkpoint inhibitors, such as antibodies that antagonize programmed cell death protein 1 (PD-1) or its ligand (PD-L1) [2]. Another effective immunotherapy is adoptive T-cell transfer, in which *ex vivo* expanded tumor-infiltrating lymphocytes (TILs), T-cells engineered with recombinant T-cell receptor (TCR), or chimeric antigen receptors (CARs) that directly target and kill cancer cells, are used to boost anti-tumor immunity [3]. Additionally, immunologic adjuvants, such as toll-like receptor (TLR) agonists, that can stimulate innate immunity, have emerged as alternative agents for cancer immunotherapy. The potential of TLR agonists for cancer treatment has been demonstrated in several studies and clinical trials [4–6]. Polyinosinic-polycytidylic acid (Poly(I : C)), a TLR3 agonist, is the most potent type I interferon (IFN) inducer [7]. TLR3 is mainly expressed in immune cells, but it is also found in tumors, such as melanoma, breast cancer and hepatocarcinoma [8–10]. As a TLR3 agonist, Poly(I : C) not only directly induces tumorous apoptosis, but also stimulates tumor cells to secrete immune factors, including IL-6, IFNα and IFNβ [10,11]. The increase in cytokine levels leads to activation of immune cells, accelerating tumor eradication [12,13]. However, the immune response rate induced by Poly(I : C) remains low in several types of malignancies and higher doses are often required to achieve the desired effect [14,15]. Furthermore, Poly(I : C) is highly toxic and therefore presents a narrow therapeutic window [16], greatly limiting clinical application of Poly(I : C)-based treatments. As such, there are two viable strategies for enhancing the therapeutic effects of immunologic adjuvants: improving efficacy and reducing toxicity.

Photodynamic therapy (PDT) is a novel non-invasive treatment option that has several advantages, including strong efficacy, target ability and negligible side effects, which has been used for treatment of tumors and other diseases [17,18]. PDT relies on photosensitizers and the use of excitation light of a specific wavelength in the presence of molecular oxygen to generate singlet oxygen and other reactive oxygen species (ROS) that induce tumor cell apoptosis and necrosis [19]. In addition, the sudden release of tumor antigens (TA) and pro-inflammatory mediators because of PDT-induced cytotoxicity can significantly initiate immune response [20,21]. Kabingu *et al.* demonstrated that local PDT not only inhibits the growth of primary tumors, but also suppresses the off-target distant tumors because of the infiltration of immune cells [22]. Therefore, PDT could potentially enhance the immune activation effect of Poly(I : C) and remedy the deficiencies mentioned above. While traditional photosensitizers (PSs) suffer from poor solubility and aggregation-caused reduction of ROS generation in aqueous media [23,24], the emergence of aggregation-induced emission luminogens (AIEgens) presents a new strategy for the construction of effective photosensitizers [25]. Their enhanced emission in the aggregate state can improve the effectiveness of PDT, especially in combination with specific targeting moieties.

To achieve tumor selectivity, nanoparticles (NPs) can be functionalized with naturally derived bio-membranes to construct biomimetic systems [26–28]. Red blood cells (RBCs) are one of the most efficient biomimetic drug delivery carriers because of their high payload efficiency, biocompatibility, degradability and deformability [29]. It has been reported that NPs coated with RBC membranes exhibit prolonged blood half-life and improved enrichment of NPs in tumors [30]. Huang's group demonstrated that RBC membrane-coated NPs exhibit the enhanced permeability and retention (EPR) effect necessary for tumor targeting [31]. Importantly, RBC biomimetic NPs are biocompatible and can be metabolized without unwanted by-products [32]. In addition, senescent or damaged RBCs are physiologically eliminated in the spleen by macrophages and dendritic cells, which can also activate the immune system [33]. Xiao *et al.* demonstrated that nanoerythrosomes derived from RBC membranes promote TA delivery to the spleen to enhance cancer immunotherapy [34]. Therefore, RBC membrane-derived NPs not only enrich in tumors, but also accumulate in the spleen because of the homing effect.

**Figure 1.**
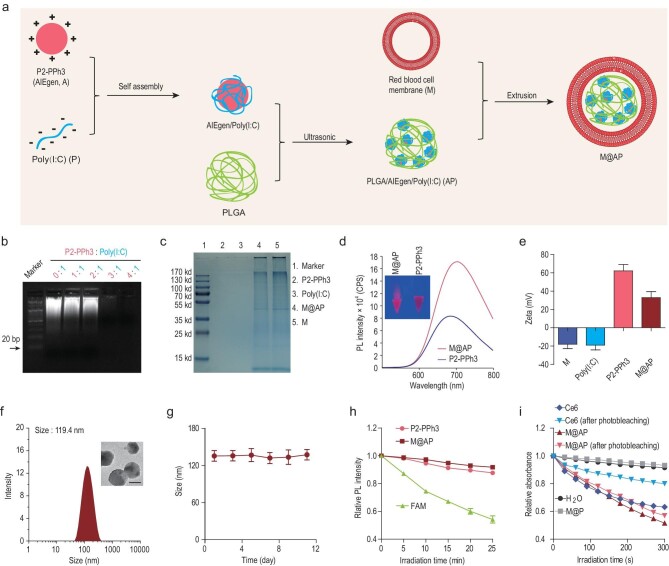
Preparation and characterization of M@AP NPs. (a) The preparation processes of M@AP NPs. (b) The optimization of the P2-PPh3 to Poly(I : C) weight ratio in the self-assembly complex of AIEgen/Poly(I : C). (c) SDS-PAGE showing protein components from M@AP compared with P2-PPh3, Poly(I : C) and M. (d) Fluorescence spectra (Ex = 506 nm) of P2-PPh3 and M@AP. (e) Zeta potential indicating the surface charges of M, Poly(I : C), P2-PPh3 and M@AP. (f) Hydrodynamic size distribution of M@AP. Insert: representative TEM image of M@AP. Scale bar: 100 nm. (g) Stability of M@AP at room temperature. (h) Photobleaching resistance of P2-PPh3, M@AP and FAM. (i) The ROS production ability of Ce6 and M@AP before and after photosensitizing measured by 9,10-anthracenediyl-bis(methylene)-dimalonic acid (ABDA) absorption. H_2_O and M@P (without P2-PPh3) are shown as control. White light irradiation (intensity: 20 mW cm^–2^) was used for photosensitizing.

Based on these features of RBC membranes, we envisioned that RBC membrane-camouflaged NPs loaded with photosensitizing AIEgen and immunologic adjuvant Poly(I : C) would strongly enhance anti-tumor immunity. In this study, we constructed such multi-functional NPs (M@AP NPs) by self-assembly of a positively charged AIE PS (i.e. P2-PPh3) and negatively charged Poly(I : C) in a poly (lactic-co-glycolic acid) (PLGA) matrix, which were then further encapsulated in an RBC membrane (Fig. 1a). Our *in vivo* results showed that the majority of the M@AP NPs are enriched in tumors, which induces tumor cell death upon PDT and promotes the release of TA to activate anti-tumor immunity. Furthermore, some of the M@AP NPs target the spleen because of the homing effect and directly activate the anti-tumor immune response. Therefore, we have demonstrated that M@AP NPs, as a combined PDT and immunotherapy agent, exhibit a direct inhibitory effect on tumors and evoke an anti-tumor immune response, representing a novel combination strategy for effective cancer treatment.

## RESULTS AND DISCUSSION

### Preparation and characterization of M@AP NPs

P2-PPh3 (structure shown in Fig. S1a) was synthesized according to our previous report [35]. P2-PPh3 is a conjugated polyelectrolyte (CPE) showing AIE properties with enhanced emission in co-solvent systems that have high fractions of tetrahydrofuran (THF), a poor solvent for P2-PPh3 (Fig. S1b). The negatively charged Poly(I : C) self-assembles with the positively charged P2-PPh3 spontaneously to form a complex. The weight ratio of P2-PPh3 to Poly(I : C) was optimized and verified by agarose gel electrophoresis. When the weight ratio of P2-PPh3 to Poly(I : C) is above 3 : 1, almost all the Poly(I : C) is involved in wrapping and thus no band for Poly(I : C) is visualized on the agarose gel (Fig. 1b). To increase the stability of the NPs, PLGA was introduced into the complex by ultrasonication to construct the PLGA/P2-PPh3/Poly(I : C) nanocore (termed AP) (Fig. S2). M@AP was then prepared by co-extrusion of AP with RBC membrane (M).

Sodium dodecyl sulfate-polyacrylamide gel electrophoresis (SDS-PAGE) was performed to verify the protein load on the NPs, confirming that M@AP indeed contains proteins from the RBC membrane, while Poly(I : C) and P2-PPh3 are protein-free (Fig. 1c). UV-Vis and fluorescence spectra showed that P2-PPh3 and M@AP have similar absorption and emission profiles (Figs S3 and 1d), with M@AP exhibiting higher fluorescence compared with that for the same amount of P2-PPh3. Comparing P2-PPh3 in DMSO solution and in the aggregated state, P2-PPh3 exhibits a higher fluorescence quantum yield of 25.5% in a DMSO/THF mixture (1 : 9 *v/v*) than in pure DMSO solution (7.3%). However, the quantum yields of M@AP in the DMSO/THF mixture and pure DMSO solution are very similar, 27.9% and 28.6%, respectively. Zeta potential measurement showed that P2-PPh3 has a large number of positive charges and that the zeta potential is lower in the resultant M@AP after assembly with Poly(I : C) and RBC membrane (Fig. 1e). The average particle size of M@AP is ∼120 nm according to transmission electron microscopy (TEM), scanning electron microscopy (SEM) and dynamic light scattering (DLS) analyses (Figs 1f and S4). Furthermore, M@AP exhibits good stability at room temperature over 10 days (Fig. 1g). Compared with conventional concentration-quenching dyes such as 6-carboxyfluorescein (FAM), P2-PPh3 and M@AP exhibit stronger photobleaching resistances (Figs 1h and S5). The ROS-production capacity of the traditional photosensitizer Ce6 is greatly reduced after photobleaching, while the same photobleaching conditions do not significantly change the photosensitivity of M@AP (Figs 1i and S6). Thus, the above results confirm that M@AP was successfully prepared and that it exhibits excellent photobleaching resistance, stability and light-induced ROS-generation capability.

### M@AP-mediated PDT drives effective anti-tumor immune responses

**Figure 2.**
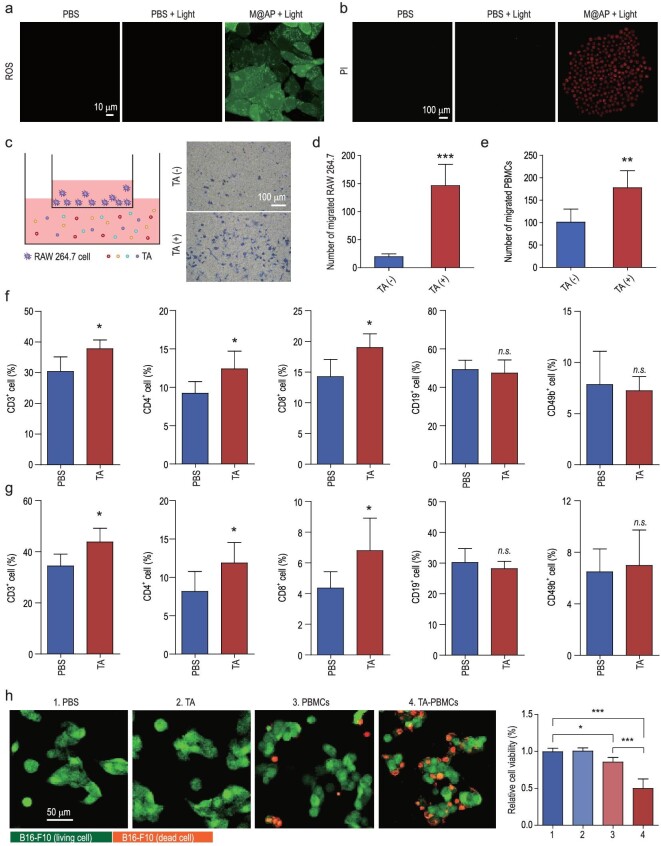
M@AP-mediated PDT promotes TA release and activates anti-tumor immunity. (a) DCFH-DA was used to detect the ROS levels in B16-F10 cells. Light intensity: 100 mW cm^–2^; irradiation time: 3 min. Ex = 488 nm; Em = 520–540 nm. Scale bar: 10 μm. (b) PI staining was performed to detect the viability of B16-F10 cells after PDT. Ex = 633 nm; Em = 640–680 nm. Scale bar: 100 μm. (c) TA released from B16-F10 cells after PDT was co-cultured with RAW 264.7 cells (Left). After 18 h co-culture, the migration of RAW 264.7 cells was detected by crystal violet staining (Right). Scale bar: 100 μm. (d) The number of RAW 264.7 cells migrated in the co-culture model. (e) The number of PBMCs migrated in the co-culture model. C57BL/6 mice were stimulated with TA released from B16-F10 cells. The proportions of CD3^+^, CD4^+^, CD8^+^, CD19^+^ or CD49^+^ cells in (f) peripheral blood and (g) spleen were detected by flow cytometry (*n* = 3). (h) PBMCs isolated from C57BL/6 mice were co-cultured with EGFP-B16-F10 cells. After 18 h co-culture, the viability of EGFP-B16-F10 cells was detected by PI staining. The green fluorescence represented EGFP-B16-F10 cells, and the red fluorescence indicated the dead cells. Scale bar: 50 μm. The data were reported as mean ± SD and analyzed by two-sided Student's t-test. ^*^P <  0.05, ^**^P <  0.01, ^***^P <  0.001.

First, we investigated the photosensitizing properties of M@AP in cells using the ROS indicator, DCFH-DA, and the dead cell indicator, propidium iodide (PI). B16-F10 cells (a mouse melanoma cell line) incubated with M@AP show red emission from the M@AP, indicating successful cell uptake of the NPs (Fig. S7). After co-incubation with DCFH-DA for 20 min, the B16-F10 cells were irradiated with white light (100 mW cm^–2^) for 3 min, and a strong green fluorescence from ROS-activated DCFH-DA and a red fluorescence from PI were observed in M@AP-treated cells, indicating that M@AP produces ROS and effectively kills cells under such conditions (Fig. 2a and b). In addition, PDT has been reported to increase the immunogenicity of tumor cells by exposing TA that can increase the efficiency of antigen cross-presentation, leading to more effective tumor immunotherapy [36–39]. Accordingly, we confirmed that PDT mediated by M@AP promotes the release of TA in B16-F10 cells using SDS-PAGE (Fig. S8). We then co-cultured the TA released from B16-F10 cells upon PDT with RAW 264.7 (a mouse macrophage cell line) (Fig. 2c). RAW 264.7 cells were placed in the upper chamber of a transwell cell migration assay apparatus and TA was added to the culture medium of the lower chamber. The migration of cells from the upper to the lower chamber depends on their migration ability under the given conditions [40]. The migration of RAW 264.7 cells was detected by crystal violet staining after 18 h of culturing, demonstrating that TA induced by PDT promotes migration of RAW 264.7 cells (Fig. 2c and d). We also co-cultured TA released from B16-F10 cells upon PDT with mouse peripheral blood mononuclear cells (PBMCs) (Fig. 2e), which include lymphocytes, monocytes and dendritic cells that play important roles in the tumor immune response processes [41]. Our results confirmed that TA also promotes the migration of PBMCs.

Next, we investigated whether TA from M@AP-treated B16-F10 cells can induce anti-tumor immune response *in vivo* by injecting the TA into C57BL/6 mice through the tail vein. According to flow cytometry analysis of the peripheral blood collected from mice, the proportions of CD3-, CD4- or CD8-positive cells (T-lymphocytes) in the TA-treated group were significantly higher than those in PBS-treated group, but there was no significant difference in CD19 (B lymphocytes)- and CD49b (natural killer (NK) cells)-positive cells (Figs 2f and S9a). Similar results were observed for the spleens of these mice, which showed that the proportions of T-lymphocytes increase after exogenous TA injection (Figs 2g and S9b). These results suggest that M@AP-mediated PDT promotes TA release from tumor cells and thus activates T-lymphocyte proliferation. To further investigate the potential cytotoxic and cytostatic activities of immune cells toward tumor cells, EGFP-B16-F10 cells were co-cultured with PBMCs isolated from the peripheral blood of mice stimulated by TA (TA-PBMCs). Imaging results showed increased numbers of dead cells, as indicated by red PI signals, in the group treated with TA-PBMCs (Fig. 2h). Together, these results suggest that the excellent PDT effect of M@AP induces tumor cells to release TA, which further activates the immune response to exert anti-tumor functions.

### Poly(I : C) treatment leads to tumor cell death *in vitro* by inducing a direct killing effect and indirect immune activation

**Figure 3.**
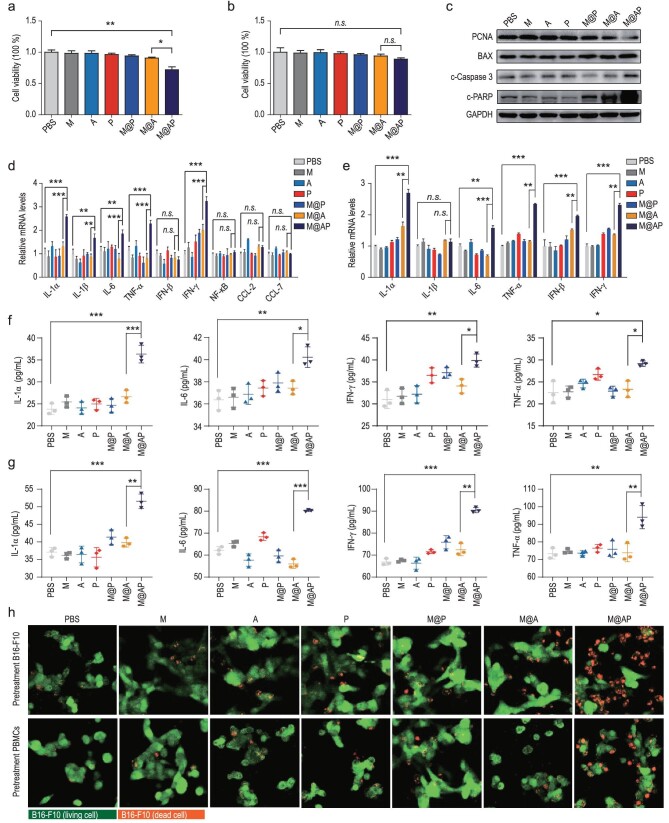
Poly(I : C) promotes tumor cell death and simultaneously activates anti-tumor immunity. (a and b) Viability of (a) B16-F10 cells and (b) RAW 264.7 cells treated with PBS, M (red blood cell membrane), A (P2-PPh3), P (Poly(I : C)), M@P (NP without P2-PPh3), M@A (NP without Poly(I : C)) and M@AP by CCK-8 kit. (c) The expression levels of PCNA, BAX, c-Caspase3, PARP and GAPDH in B16-F10 cells treated with M@AP and controls detected by western blot. (d and e) The mRNA levels of immune factors in (d) B16-F10 and (e) PBMCs treated with M@AP and controls detected by qRT-PCR. (f and g) The protein levels of immune factors in (f) B16-F10 and (g) PBMCs treated with M@AP and controls detected by ELISA. (h) Upper: EGFP-B16-F10 cells were pretreated with PBS, M, A, P, M@P, M@A or M@AP, and then co-cultured with untreated PBMCs for 18 h. Lower: PBMCs were pretreated with PBS, M, A, P, M@P, M@A or M@AP, and then co-cultured with untreated EGFP-B16-F10 for 18 h. Dead EGFP-B16-F10 cells were shown in orange (merged green EGFP and red PI signals). Scale bar: 100 μm. The data were reported as mean ± SD and analyzed by two-sided Student's t-test (*n* = 3). ^*^P <  0.05, ^**^P <  0.01, ^***^P <  0.001, *n.s*. not significant.

To validate the role of Poly(I : C) in M@AP NPs, we first constructed M@AP-FITC NPs using FITC-tagged Poly(I : C) (Fig. S10). After 8 h incubation with M@AP-FITC, fluorescent signals from both Poly(I : C)-FITC and P2-PPh3 were detected in the cells (Fig. S11). Then, we prepared M@A NPs using the same procedure but without adding Poly(I : C). The effects of different NPs (without light irradiation) on the cell viability of B16-F10 cells were determined using Cell Counting Kit-8 (CCK-8) tests, from which results revealed that only M@AP NPs can induce significantly lower cell viability (Fig. 3a). Poly(I : C)-mediated cell death combined with P2-PPh3-mediated PDT can significantly enhance the cytotoxicity of M@AP (Fig. S12). In the concentration range 0–40 μg/mL, M@AP has no toxic effect on RAW 264.7 cells (Figs 3b and S13). To investigate the cytotoxic mechanism of M@AP towards B16-F10 cells, western blotting was used to reveal the expression of biomarkers related to cell proliferation (PCNA) and apoptosis (BAX, c-Caspase3 and c-PARP). The expression of PCNA in B16-F10 cells was lower in the M@AP group, while those of BAX, c-Caspase3 and c-PARP increased significantly after M@AP treatment (without light irradiation) (Figs 3c and S14), indicating that M@AP treatment effectively inhibits tumor cell proliferation and promotes tumor cell apoptosis.

In addition, the presence of M@AP has been observed to significantly up-regulate the mRNA expression of a series of immune factors, including IL-1α, L-1β, IL-6, TNF-α and IFN-γ, in B16-F10 cells (Fig. 3d). Such activation of immune inflammation response in cancer cells has been attributed to induction of the nuclear transcription factor kappa B (NF-κB) pathway [42]. Importantly, several studies have demonstrated that Poly(I : C) can enhance anti-tumor responses by mediating the activation of immune cells [43]. Accordingly, we studied the immune-activation effects of M@AP on RAW 264.7 cells, PBMCs and mouse bone marrow-derived macrophages (BMDMs). Confocal laser scanning microscopy (CLSM) imaging confirmed that M@AP can enter RAW 264.7 cells (Fig. S15). Upon M@AP treatment, we observed the up-regulation of immune factors (IL-1α, IL-6, TNF-α and IFN-γ) at the mRNA levels not only in RAW 264.7 cells (Fig. S16), but also in PBMCs (Fig. 3e) and BMDMs (Fig. S17). Furthermore, M@AP incubation promoted the release of IL-1α, IL-6, TNF-α and IFN-γ from B16-F10 cells (Fig. 3f), PBMCs (Fig. 3g) and BMDMs (Fig. S18). Collectively, the above results confirm the role of M@AP in promoting immune activation.

To further investigate the immune activation induced by M@AP on tumor cells, we first pretreated EGFP-B16-F10 with PBS, M, A, P, M@P, M@A or M@AP, and then co-cultured them with untreated PBMCs for 18 h (Fig. 3h, upper). We also pretreated PBMCs with M@AP or the controls, and then co-cultured them with untreated EGFP-B16-F10 (Fig. 3h, lower). In both sets of experiments, we observed that the number of dead B16-F10 cells (indicated by merged green and red signals) increased in the M@AP-treated groups, especially in the EGFP-B16-F10 group pretreated with M@AP (Figs 3h and S19). Thus, we confirmed that, alongside its PDT activity, M@AP has a direct anti-tumor effect and is also capable of activating anti-tumor immunity to kill tumor cells through an indirect method.

### Metabolism and anti-tumor effect of M@AP *in vivo*

**Figure 4.**
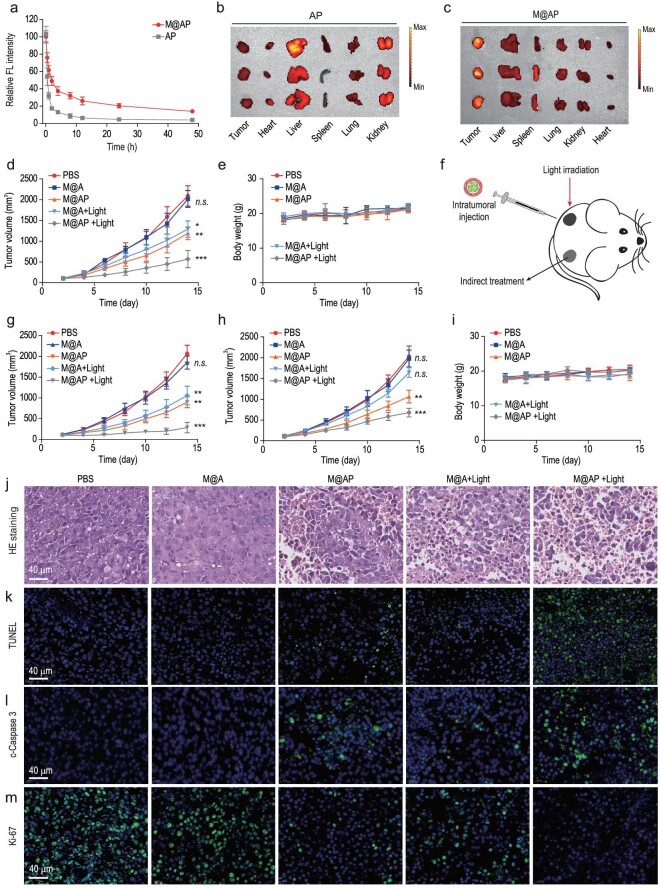
The metabolism and anti-tumor effect of M@AP *in vivo*. (a) Pharmacokinetics of M@AP and AP (PLGA/AIEgen/Poly(I : C)). (b and c) The biodistribution of (b) AP and (c) M@AP in the tumor, heart, liver, spleen, lungs and kidneys. The color spectral gradient bars represented the fluorescence intensity. (d) Growth kinetics of B16-F10 subcutaneous tumors (unilateral) treated with PBS, M@A, M@AP, M@A +Light and M@AP +Light, respectively (*n* = 5). (e) Body weight changes of unilateral B16-F10 tumor-bearing mice during treatment (*n* = 5). (f) Bilateral B16-F10 tumor-bearing mice model: B16-F10 cells were inoculated on the left and right backs, respectively (*n* = 5). Intratumoral injection and light irradiation were performed only on the left tumor. The growth kinetics of the (g) left and (h) right tumors. (i) Body weight changes of bilateral B16-F10 tumor-bearing mice during treatment (*n* = 5). (j) Representative H&E and (k) TUNEL staining of tumor tissue in unilateral B16-F10 tumor-bearing mice model. The expression levels of (l) c-Caspase3 and (m) Ki-67 in tumor tissues were indicated by green fluorescence (*n* = 5). Blue fluorescence is the nucleus stained by 4',6-diamidino-2-phenylindole (DAPI). The data are shown as mean ± SD and analyzed by two-sided Student's t-test. ^*^P <  0.05, ^**^P <  0.01, ^***^P <  0.001, *n.s*. not significant.

B16-F10 tumor-bearing mice were used to investigate the distribution and anti-tumor effect of the NPs *in vivo*. We confirmed that M@AP can be injected intravenously without inducing hemolysis (Fig. S20). The pharmacokinetics of M@AP and AP (NPs without the RBC membrane) are shown in Fig. 4a. M@AP remains circulating in the blood for a longer time without non-specific scavenging compared with AP, which indicates that the RBC membrane coating prolongs the circulation. Furthermore, to investigate the localization of NPs *in vivo*, the biodistributions of AP and M@AP in the tumors and organs were detected using an *in vivo* imaging system (IVIS^®^) (Fig. 4b and c). The results show that AP mainly concentrates in the liver and kidneys, while M@AP has a propensity to accumulate in the tumor and spleen (Fig. S21). Aged or damaged RBCs are physiologically eliminated by macrophages or dendritic cells in the spleen [34] and this homing effect makes M@AP target the spleen (Figs 4b and c, and S21). In addition, because of the prolonged blood circulation of RBC membrane-camouflaged NPs, the EPR effect of the NPs is enhanced and the tumor-targeting ability of M@AP NPs is thus strengthened. As a result, the enrichment of M@AP in the tumor and spleen has a synergistic effect involving both immune activation and anti-tumor activity.

To investigate the anti-tumor efficacy of the NPs *in vivo*, unilateral B16-F10 tumor-bearing mice with tumor volumes of ∼100 mm^3^ were randomly divided into five groups: PBS (Light −), M@A (Light −), M@AP (Light −), M@A (Light +) and M@AP (Light +). As shown in Fig. 4d, tumor growth values in the M@AP (Light −), M@A (Light +) and M@AP (Light +) groups are lower compared with those in the PBS (Light −) and M@A groups. The inhibition degree for the M@AP (Light +) group is higher than those for the M@AP (Light −) and M@A (Light +) groups, which indicates that AIEgen-mediated PDT can enhance Poly(I : C)-mediated immunotherapy to inhibit tumor growth. Furthermore, the body weights of the mice show almost no difference between five treatment groups, indicating the excellent biocompatibility of the NPs (Fig. 4e).

To explore the efficacy of M@AP in the treatment of tumors, a bilateral B16-F10 tumor-bearing mouse model was constructed by inoculating B16-F10 cells on both the left and right back tissues of mice. The tumors on the left side were injected with NPs and received light irradiation (Fig. 4f), and the growth kinetics of the left and right tumors were monitored. As shown in Fig. 4g, the tumors on the left are significantly inhibited in the M@AP (Light +) group compared with those in the M@AP (Light −) and M@A (Light +) groups, which indicates that the combination of PDT and Poly(I : C) has a better anti-tumor effect than each individually. For the M@A (Light +) group, tumor growth is slightly inhibited on the non-treatment side (no drug injection and no light irradiation), but the difference is not statistically significant (*P* > 0.05), indicating that the immune effect induced by PDT is insufficient to inhibit growth of the adjacent tumor. Interestingly, the non-treatment side tumor in the M@AP (Light −) group is significantly inhibited (*P* < 0.05), and the treatment effect for the non-treatment side tumor in the M@AP (Light +) group is the highest (*P* < 0.001) (Fig. 4h). We attributed this observation to the enhanced immune activation induced by the synergetic effect of AIEgen-mediated PDT and Poly(I : C) (Fig. 4h). Similarly, no weight loss is observed for any of the groups (Fig. 4i), which indicates that the NPs are safe *in vivo*. Collectively, these results suggest that M@AP kills tumor cells by both directly and indirectly activating immunity.

To further explore the anti-tumor mechanism of the NPs, we examined the histopathology of the tumor tissues using H&E staining. The results reveal that the tumor tissues in the PBS and M@A (Light −) groups are compact, whereas those in the M@AP (Light −), M@A (Light +) and M@AP (Light +) groups are sparse with a large number of vacuolar necrotic cells (Fig. 4j). Furthermore, terminal deoxynucleotidyl transferase-mediated dUTP-biotin nick end labeling (TUNEL) staining shows that apoptotic tumor cells are abundant in the M@AP (Light +) group, while only a few apoptotic cells are observed in the M@AP (Light −) and M@A (Light +) groups and almost no apoptotic cells are observed in the PBS and M@A (Light −) groups (Figs 4k and S22a). In addition, it is generally believed that c-Caspase 3 is the most important terminal splicing enzyme in the apoptosis process [44]. Our results show that the expression of c-Caspase3 is significantly increased in the M@AP (Light −), M@A (Light +) and M@AP (Light +) groups, indicating that more tumor cells undergo apoptosis in these groups (Figs 4l and S22b). Ki-67 is a cell-proliferation-related gene that reflects the proliferative capability of tumor cells [45]. We measured the expression of Ki-67 in the tumors, and the results reveal that the densities of Ki-67-positive cells in the tumors of the M@AP (Light −), M@A (Light +) and M@AP (Light +) groups are lower than those in the PBS (Light +) and M@A (Light −) groups (Figs 4m and S22c). Collectively, these results demonstrate that combining PDT with Poly(I : C) treatment is an effective way to inhibit the proliferation and promote the apoptosis of tumor cells.

### M@AP treatment enhances anti-tumor immune activity *in vivo*

**Figure 5.**
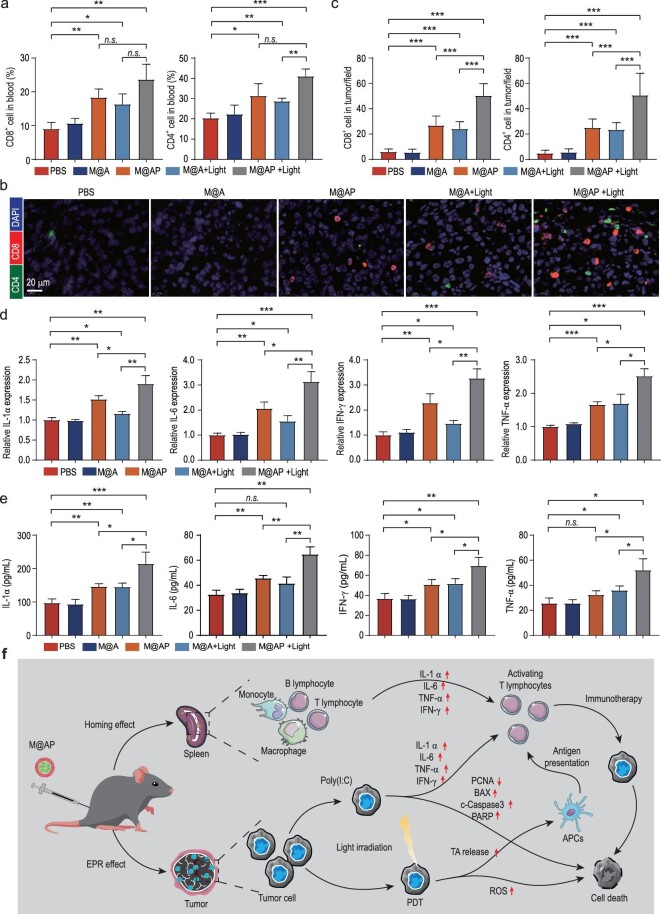
Anti-tumor mechanism of M@AP *in vivo*. (a) The proportion of CD8^+^ and CD4^+^ cells in the peripheral blood of B16-F10 tumor-bearing mice. (b and c) The abundance of CD4^+^ and CD8^+^ cells in tumor tissue as revealed by CLSM (b) images and (c) quantification. (d) The expression levels of IL-1α, IL-6, IFN-γ and TNF-α mRNA in spleen detected by qRT-PCR. (e) The expression levels of IL-1α, IL-6, IFN-γ and TNF-α in plasma detected by ELISA. (f) The proposed anti-tumor mechanism of M@AP. EPR effect: enhanced permeability and retention effect; PDT: photodynamic therapy; ROS: reactive oxygen species; TA: tumor antigen; APCs: antigen presenting cells. The data were reported as mean ± SD and analyzed by two-sided Student's t-test (*n* = 3). ^*^P <  0.05, ^**^P <  0.01, ^***^P <  0.001, *n.s*. not significant.

We then investigated the mechanism by which M@AP NPs activate the immune system against tumors *in vivo*. T-lymphocytes are key mediators of tumor destruction and their specificity for tumor-expressed antigens is of paramount importance [46]. CD8^+^ T-cells are the preferred tool for investigating anti-tumor activities as they perform tumorous antigen presentations, and CD4^+^ T-cells are also necessary to support the normal functioning of CD8^+^ T-cells [47]. As shown in Figs 5a and S23, the proportions of CD4^+^ T-cells and CD8^+^ T-cells in the peripheral blood from mice are significantly higher in the M@AP (Light −), M@A (Light +) and M@AP (Light +) groups compared with the control group, with the M@AP (Light +) group showing the most obvious increase. Furthermore, as analyzed by CLSM imaging (Figs 5b and c, and S24), the number of CD4^+^ T-cells and CD8^+^ T-cells is also increased in the tumor tissues, suggesting that the combination of PDT and Poly(I : C) promotes tumor-specific T-cell responses. In addition, tumor-associated macrophages (TAMs) are the major infiltrating leukocytes of the tumor microenvironment, among which M1 macrophages are reported to phagocytose tumor cells [48]. Our results showed that levels of M1 macrophages (F4/80^+^, CD11b^+^ and CD86^+^) are significantly increased in tumor sections from M@AP-treated groups, especially the M@AP (Light +) group (Fig. S25), suggesting that M@AP promotes the differentiation of macrophages into the M1 type. In addition, we also investigated the population of regulatory T (Treg) cells (FOXP3^+^ and CD4^+^), which play important roles in suppressing anticancer immunity. As shown in Fig. S26, the absolute number of Treg cells in the tumor tissues is relatively low in all groups (PBS, M@A, M@AP, M@A (+Light) and M@AP (+Light) groups). But the proportions of Treg cells in the M@AP, M@A (+Light) and M@AP (+Light) groups are decreased, among which the degree of decline in the M@AP (+Light) group is the most obvious.

Furthermore, aged or damaged RBCs are eliminated by scavenger cells such as macrophages and dendritic cells in the spleen [34]. Poly(I : C) loaded in RBC membranes specifically accumulates in the spleen and stimulates immune cells to produce immune factors such as IL-1α, IL-1β, IL-6, IL-12, IL-23, TNF-α, IFN-β and IFN-γ [49]. Several cytokines released from immune cells then suppress tumor cell growth by direct anti-proliferative or pro-apoptotic activity, or by indirectly stimulating the cytotoxic activity of immune cells against tumor cells [50]. Therefore, we used real-time quantitative PCR (qRT-PCR) to detect the expression of cytokines in the spleen, revealing that IL-1α, IL-6, IFN-γ and TNF-α are highly expressed in the M@AP (Light −), M@A (Light +) and M@AP (Light +) groups (Fig. 5d). Next, the levels of systemic cytokines in the peripheral blood were determined after treatment. Remarkably, the ELISA results also demonstrate increased concentrations of IL-1α, IL-6, IFN-γ and TNF-α in the sera of mice in the M@AP (Light −), M@A (Light +) and M@AP (Light +) groups (Fig. 5e). The levels of immune factors both in the spleen and peripheral blood indicate that PDT or Poly(I : C) can activate anti-tumor immunity to some extent, and that the combination of PDT and Poly(I : C) can enhance anti-tumor immunity. To assess the safety of NPs, H&E staining of the organs in the different groups was performed. As shown in Fig. S27, there are no pathological changes in the heart, liver, spleen, lung or kidney tissues for each group of mice after injection with different types of NPs. In summary, the above results indicate that M@AP NPs, as a combination of PDT and immunotherapy, exhibit a direct inhibitory effect on tumors and evoke a robust anti-tumor immune response (Fig. 5f).

### Application of M@AP to B16-F10 lung metastasis therapy

**Figure 6.**
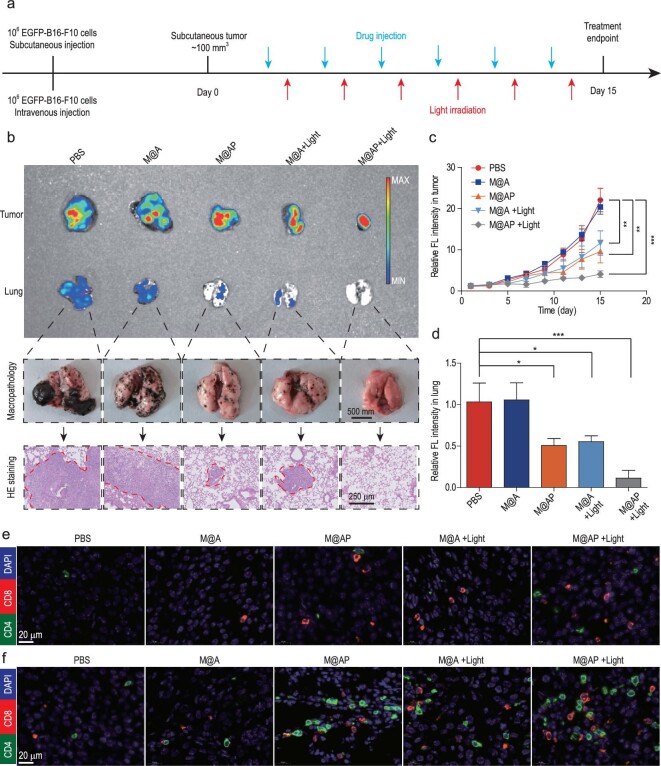
The therapeutic effect of M@AP on B16-F10 lung metastasis mice. (a) The objective to construct EGFP-B16-F10 subcutaneous tumor and lung metastasis mouse models and the treatment process. (b) Lung metastases were observed by (top) IVIS Spectrum imaging system, (middle) naked eye and (bottom) microscope. (c) Growth kinetics of the EGFP-B16-F10 subcutaneous tumors. (d) Quantitative analysis of lung metastases by IVIS Spectrum imaging system. (e and f) The abundance of CD4^+^ and CD8^+^ cells in the (e) tumors and (f) lungs. Scale bar: 20 μm. The data were reported as mean ± SD and analyzed by two-sided Student's t-test (*n* = 3). ^*^P <  0.05, ^**^P <  0.01, ^***^P <  0.001.

Tumor metastasis has always been a problem in cancer therapy. Accordingly, the promising anti-tumor activity of M@AP promoted us to explore its therapeutic potential for lung metastasis. The therapeutic process is shown in Fig. 6a. C57BL/6 mice with subcutaneous tumors and lung metastasis were prepared by intravenous and subcutaneous injection with EGFP-B16-F10 cells. When the volume of the subcutaneous tumor reached 100 mm^3^, randomized mice were treated with PBS (Light −), M@A (Light −), M@AP (Light −), M@A (Light +) or M@AP (Light +). The interval of drug injection and light irradiation is shown in Fig. 6a. On day 15, the treatment was stopped and the tumors were extracted for analysis. In M@AP (Light −), M@A (Light +) and M@AP (Light +) treatment groups, the volume of the subcutaneous tumors is significantly reduced and more significantly, lung metastasis is also less profound than in other groups, as revealed by IVIS^®^ imaging, observation with the naked eye and H&E staining (Fig. 6b). In particular, H&E staining of lung sections revealed that M@AP (Light +) treatment is superior, even compared with M@AP (Light −) or M@A (Light +) treatments, with better anti-metastasis activity and fewer metastatic tumor deposits in the lung. Further analysis showed strong EGFP signals for the tumor and lung tissues in PBS and M@A (Light −) groups, weak EGFP signals in M@AP (Light −) and M@A (Light +) groups, and almost no EGFP signal in the M@AP (Light +) group (Fig. 6c and d). Weaker EGFP signals indicate lower viabilities for B16-F10 tumor cells. Therefore, these results confirm the excellent therapeutic benefits of M@AP against lung metastasis. To further investigate the therapeutic mechanism of M@AP towards lung metastasis, immune cells in the subcutaneous tumor (Figs 6e and S28) and lung (Figs 6f and S29) metastasis were examined by immunofluorescence staining. The results indicated that systemic administration of M@AP (Light +) significantly increases the numbers of CD4^+^ T-cells and CD8^+^ T-cells in the tumor (Figs 6e and S28) and inhibits lung metastasis (Figs 6f and S29). Collectively, the above results show that M@AP has a therapeutic effect on lung metastasis by activating anti-tumor immunity.

## CONCLUSION

In this study, we developed a multi-functional nanoplatform for delivering immunologic adjuvants for synergistic anti-tumor photodynamic immunotherapy. The M@AP nanoplatform consists of a complex comprising an AIE-conjugated polymer as a photosensitizer and Poly(I : C) as an immunologic adjuvant, a PLGA matrix and an RBC membrane shell. *In vivo*, M@AP NPs are mainly enriched in tumor tissues because of the ERP effect, but they also accumulate in the spleen through the homing effect of the RBC membrane. The latter activates immune cells in the spleen to strengthen anti-tumor immunity. Under white light irradiation on the tumor region, the AIE photosensitizer induces tumor cell death by generating intracellular ROS, which further promotes the release of TA and activates the immune response with a synergistic effect of Poly(I : C). Quantitative analysis revealed that M@AP NPs simultaneously kill local cancer cells directly and stimulate the immune cells to release cytokines that present cytotoxicity against tumor cells. We further applied the system in a lung metastasis mouse model and demonstrated the excellent anti-metastasis capability of our approach, which provides a potential treatment strategy for preventing tumor recurrence and metastasis.

## Supplementary Material

nwab039_Supplemental_File
