# Correction to: Red blood cell membrane-camouflaged nanoparticles loaded with AIEgen and Poly(I:C) for enhanced tumoral photodynamic-immunotherapy

**DOI:** 10.1093/nsr/nwaf324

**Published:** 2025-09-08

**Authors:** Jun Dai, Meng Wu, Quan Wang, Siyang Ding, Xiaoqi Dong, Liru Xue, Qingqing Zhu, Jian Zhou, Fan Xia, Shixuan Wang, Yuning Hong

**Affiliations:** Department of Obstetrics and Gynecology, Tongji Hospital, Tongji Medical College, Huazhong University of Science and Technology, Wuhan 430032, China; Department of Obstetrics and Gynecology, Tongji Hospital, Tongji Medical College, Huazhong University of Science and Technology, Wuhan 430032, China; Engineering Research Center of Nano-Geomaterials of Ministry of Education, Faculty of Materials Science and Chemistry, China University of Geosciences, Wuhan 430074, China; Department of Chemistry and Physics, La Trobe Institute for Molecular Science, La Trobe University, Melbourne 3086, Australia; Engineering Research Center of Nano-Geomaterials of Ministry of Education, Faculty of Materials Science and Chemistry, China University of Geosciences, Wuhan 430074, China; Department of Obstetrics and Gynecology, Tongji Hospital, Tongji Medical College, Huazhong University of Science and Technology, Wuhan 430032, China; Department of Obstetrics and Gynecology, Tongji Hospital, Tongji Medical College, Huazhong University of Science and Technology, Wuhan 430032, China; College of Material, Chemistry and Chemical Engineering, Hangzhou Normal University, Hangzhou 311121, China; Engineering Research Center of Nano-Geomaterials of Ministry of Education, Faculty of Materials Science and Chemistry, China University of Geosciences, Wuhan 430074, China; Department of Obstetrics and Gynecology, Tongji Hospital, Tongji Medical College, Huazhong University of Science and Technology, Wuhan 430032, China; Department of Chemistry and Physics, La Trobe Institute for Molecular Science, La Trobe University, Melbourne 3086, Australia

In the Fig. 3 of ‘Red blood cell membrane-camouflaged nanoparticles loaded with AIEgen and Poly(I:C) for enhanced tumoral photodynamic-immunotherapy’ (*National Science Review*, Volume 8, Issue 6, 2021, nwab039, https://doi.org/10.1093/nsr/nwab039), the image showing M@P-pretreated PBMCs co-cultured with B16-F10 was provided incorrectly (Fig. 3h). The corrected version of Fig. 3 is presented below.

**Figure 3.**
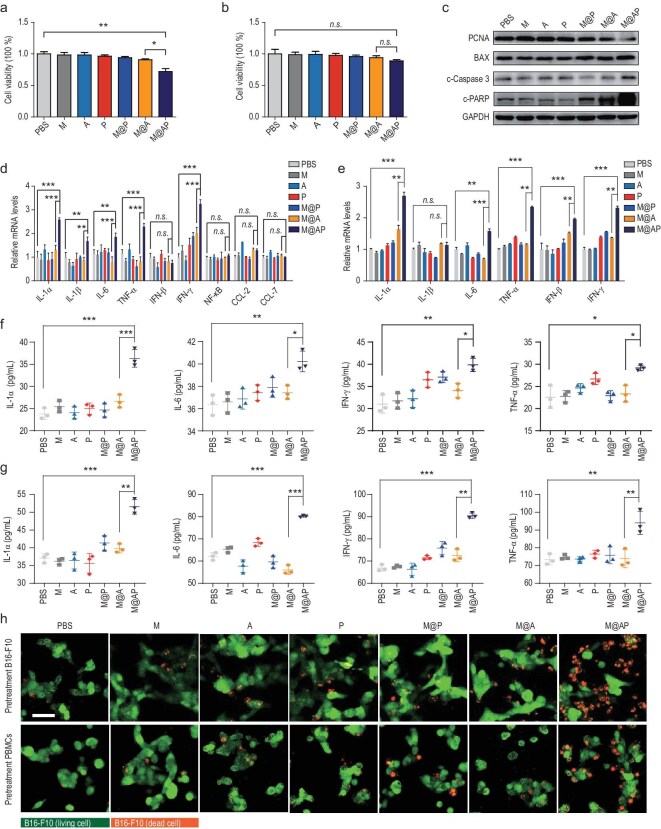
Poly(I:C) promotes tumor cell death and simultaneously activates anti-tumor immunity. (a, b) Viability of (a) B16-F10 cells and (b) RAW 264.7 cells treated with PBS, M (red blood cell membrane), A (P2-PPh3), P (Poly(I:C)), M@P (NP without P2-PPh3), M@A (NP without Poly(I:C)) and M@AP by CCK-8 kit. (c) The expression levels of PCNA, BAX, c-Caspase3, PARP and GAPDH in B16-F10 cells treated with M@AP and controls detected by western blot. (d, e) The mRNA levels of immune factors in (d) B16-F10 and (e) PBMCs treated with M@AP and controls detected by qRT-PCR. (f, g) The protein levels of immune factors in (f) B16-F10 and (g) PBMCs treated with M@AP and controls detected by ELISA. (h) Upper: EGFP-B16-F10 cells were pretreated with PBS, M, A, P, M@P, M@A or M@AP, and then co-cultured with untreated PBMCs for 18 h. Lower: PBMCs were pretreated with PBS, M, A, P, M@P, M@A or M@AP, and then co-cultured with untreated EGFP-B16-F10 for 18 h. Dead EGFP-B16-F10 cells were shown in orange (merged green EGFP and red PI signals). Scale bar: 100 μm. The data were reported as mean ± SD and analyzed by two-sided Student's *t*-test (n = 3). *P < 0.05, **P < 0.01, ***P < 0.001, *n.s*. not significant.

