# Supplementary material for: Red blood cell membrane-camouflaged nanoparticles loaded with AIEgen and Poly(I : C) for enhanced tumoral photodynamic-immunotherapy
Source: Natl Sci Rev. 2021 Mar 3;8(6):nwab039. doi: 10.1093/nsr/nwab039 (PMC8288176; doi:10.1093/nsr/nwab039)
Supplement: nwab039_Supplemental_File [file nwab039_supplemental_file.docx]

**Supporting Information**

**Red blood cell** **membrane-camouflaged nanoparticles loaded with dual AIEgen and Poly(I:C) for enhanced tumoral photodynamic-immunotherapy**

Jun Dai ^1†^, Meng Wu ^1†^, Quan Wang ^2^, Siyang Ding ^3^, Xiaoqi Dong ^2^, Liru Xue ^1^, Qingqing Zhu ^1^, Jian Zhou ^4^, Fan Xia ^2*^, Shixuan Wang ^1*^, Yuning Hong ^3*^

^1^ Department of Obstetrics and Gynecology, Tongji Hospital, Tongji Medical College, Huazhong University of Science and Technology, Wuhan 430032, China

^2^ Engineering Research Center of Nano-Geomaterials of Ministry of Education, Faculty of Materials Science and Chemistry, China University of Geosciences, Wuhan 430074, China

^3^ Department of Chemistry and Physics, La Trobe Institute for Molecular Science, La Trobe University, Melbourne, Victoria 3086, Australia

^4^ College of Material, Chemistry and Chemical Engineering, Hangzhou Normal University Hangzhou 311121, China

Corresponding authors: [y.hong@latrobe.edu.au](mailto:y.hong@latrobe.edu.au) (Y.H.); [shixuanwang@tjh.tjmu.edu.cn](mailto:shixuanwang@tjh.tjmu.edu.cn) (S.W.); [xiafan@cug.edu.cn](mailto:xiafan@cug.edu.cn) (F.X.)

**Materials and methods**

**Materials**.

Poly(I:C) and Poly(I:C)-FITC were purchased from InvivoGen (USA). PLGA [Poly (lactic-co-glycolic acid) (lactide, glycolide (50:50); MW, 5000-15000)] was purchased from Sigma-Aldrich (USA). Polycarbonate membrane (0.1 μm) was purchased from Whatman (USA). Amicon® Ultra Centrifugal Filters (UFC8050) was purchased from Amicon (USA). 2′,7′-Dichlorofluorescin diacetate (DCFH-DA) and Chlorin e6 (Ce6) were provided by Yeasen Co. Ltd. (China). 9,10-Anthracenediyl-bis(methylene)dim alonic acid (ABDA) was provided by Sigma-Aldrich (USA). Propidium iodide (PI) and Cell Counting Kit-8 (CCK8) were purchased from Beyotime Biotechnology (China). Transwell chamber was purchased from Corning (USA). Anti-CD3, CD4, CD8, CD19 and CD49b antibodies for flow cytometry were purchased from Biolegend Co. Ltd (China). Anti-Ki-67, c-Caspase3, CD11b and F4/80 antibodies were purchased from Biossci (China). Antibody against CD86 was purchased from CST (USA). Anti-PCNA, BAX, c-Caspase3, c-PARP and GAPDH antibodies used in western blot were purchased from Abclonal (China). The enhanced Western Bright ECL was purchased from Advansta (China). Mouse IL-1α, IL-6, IFN-γ and TNF-α ELISA kits were purchased from NEOBIOSCIENCE (China). The RNAiso plus for RNA extraction was purchased from Takara Biomedical Technology (China). HiScript II 1st Strand cDNA Synthesis Kit and SYBR qPCR Mix for PCR were purchased from Vazyme (China). Mouse PBMCs isolation kit was purchased from Solarbio Science & Technology (China). Mouse macrophage colony-stimulating factor (M-CSF) was purchased from PEPROTECH (China).

**Preparation of M@AP nanoparticles**.

The membrane of red blood cells (RBCs) was extracted from the whole blood of C57BL/6 mice, which was taken and added into phosphate-buffered saline PBS) (1×, pH =7.2) containing EDTA (1 mM). RBCs were isolated from the whole blood through centrifugation (2,000 rpm, 5 min). In order to obtain the RBC membrane, the hypotonic method was carried out according to the previous literature [1]. Firstly, the RBCs were mixed with deionized water containing EDTA (1 mM), followed by gently shaking for 5 min. The resultant mixture was then centrifuged (4,000 rpm, 10 min), and the supernatant was collected for continued centrifuging (148,000 rpm, 20 min) at 4 °C. Secondly, deionized water containing EDTA (1 mM) was poured into the sediment collected from above centrifuging steps, and the mixture was mixed by ultraphonic for 10 s and then centrifuged again (148,000 rpm, 20 min) at 4 °C. To complete the removal of hemoglobin, we repeated the second step twice by using deionized water containing EDTA (1 mM). The resultant pellets after washing were used as RBC membrane vesicles.

The preparation of M@AP is referred to the previous studies [2,3]. Briefly, to 2.0 mg P2-PPh3 dissolved in 0.4 mL of DMSO, 0.3 mL of Poly(I:C) (dissolved in deionized water, 2.0 mg/mL) was added dropwise under 3 min sonication (power, 39 W; frequency, 20 kHz) to form the P2-PPh3/ Poly(I:C) complex. Subsequently, to 4.0 mg PLGA dissolved in 2.0 mL of acetonitrile, P2-PPh3/ Poly(I:C) complex was added dropwise under 3 min sonication (power, 39 W; frequency, 20 kHz) to form the PLGA/P2-PPh3/Poly(I:C) (AP) core. Next, 2.0 mg RBC membrane was dissolved in 1.0 mL deionized water under ultrasound (power, 39 W; frequency, 20 kHz). To construct M@AP, RBC membrane vesicles and AP cores were coextruded through a polycarbonate membrane (0.1 μm). Organic solvents (DMSO and acetonitrile) were then removed through a centrifugal filter and the nanoparticles were diluted in DEPC treated water. Others, such as M@P (without P2-PPh3) and M@A (without Poly(I:C)), were prepared following the same processes.

**Fluorescence measurement**

The fluorescence of NPs was measured by an Agilent Cary Eclipse Fluorescence Spectrophotometer. Fluorescence quantum yields were measured using a Hamamatsu absolute PL quantum yield spectrometer C11347 Quantaurus_QY.

**Transmission electron microscopy (TEM)**.

Each sample (0.2 mg/mL, 10 μL) was dropped on a copper mesh, respectively. The phosphotungstic acid solution (10 μL) was dropped on this copper mesh to stain the samples for 3 min. After washing the copper mesh with deionized water for 30 seconds, most of the phosphotungstic acid solution was removed. The morphologies of different samples were observed by TEM (FEI Tecnai G2 20).

**Scanning electron microscopy (SEM)**

The M@AP NPs (0.2 mg/mL, 100 μL) slowly drips onto the silicon chip. After drying under vacuum, the silicon chip was sputter-coated with 5 nm of Pt prior to SEM imaging. The morphology of the M@AP NPs was measured by field SEM (FESEM, SU8010, Hitachi).

**Agarose gel electrophoresis**

3 % agarose gel was used to detect the weight ratio of Poly(I:C) and P2-PPh3. 3 % agarose (dissolved in 1× TBE buffer) was heated to complete solution. Then, ethidium bromide solution (final concentration 0.5 μg/mL) was added and fully mixed. Poly(I:C) and P2-PPh3 mixtures with different weight ratios (P2-PPh3 : Poly(I:C) =0:1, 1:1, 2:1, 3:1 and 4:1, respectively) were used for agarose gel electrophoresis. The current of electrophoresis was 100 mA and the time was 30 min. After electrophoresis, the bands of Poly(I:C) were observed under UV light (Tanon-5200 Multi, China).

**Sodium dodecyl sulfate**-**polyacrylamide gel electrophoresis (SDS-PAGE)**

B16-F10 cells were replaced with serum-free medium before photodynamic therapy. The cells treated with M@AP were irradiated with white light (100 mW cm^-2^) for 3 min, then the cell culture medium were collected. Cell culture medium (contains all proteins) were separated on SDS-PAGE gels (concentration 10 %). The separated proteins were then transferred to the nitrocellulose (NC) membrane and incubated with Coomassie brilliant blue. The proteins were visualized with the Western Blot exposure (Bio-Rad, USA).

**Dynamic light scattering (DLS)**

The hydrodynamic size distribution of the NPs were detected by Nano-ZS ZEN3690 (Malvern Instruments) at 25 °C in PBS buffer. The concentration of NPs is 0.2 mg/mL.

**Zeta potential**

Nano-ZS ZEN3690 (Malvern Instruments) was used to detect the zeta potential of NPs. All the results were measured in PBS buffer at 25 °C. The concentration of NPs is 0.2 mg/mL.

**Photobleaching resistance experiment**

For the photobleaching resistance experiment, samples were irradiated under white light (100 mW cm^-2^) for 25 min, and the fluorescence intensity of each sample was detected every 5 min during irradiation. Fluorescence spectrum was measured by Edinburgh FS5 Fluorescence Spectrophotometer (Edinburgh Instruments, U.K.).

**Detection of reactive oxygen species (ROS) in solution**

The ROS production in the H_2_O, M@P (without P2-PPh3), Ce6, Ce6 (after photobleaching), M@AP, and M@AP (after photobleaching) groups were measured by 9,10-anthracenediyl-bis(methylene)-dimalonic acid (ABDA) under white light irradiation (20 mW cm^-2^). UV spectrum and fluorescence spectrum were measured by Shimadzu UV-2600 spectrometer (Shimadzu, Japan).

**Cell culture**

The B16-F10 and RAW 264.7 cell lines were purchased from American Tissue Culture Collection (ATCC) and cultured according to the guidelines. The cells were used for the experiments within the first 20 passages.

**Generation of mouse bone marrow-derived macrophages (BMDMs)**

The femurs and tibias of C57BL/6 mice (female, 6-8 weeks) were dissected and flushed with PBS using a 27 G syringe to obtain bone marrow cells. Next, the bone marrow cells were cultured in Dulbecco Modified Eagle Medium (DMEM) medium supplemented with 10 % FBS, 1 % penicillin and streptomycin and incubated with 20 ng/mL recombinant M-CSF in a 5 % CO2 humidity incubator at 37 °C for 5 days. Non-adherent cells were discarded, and adherent cells were collected for further experiments.

**Isolation of mouse peripheral blood mononuclear cells (PBMCs)**

PBMCs from mouse blood were isolated using an isolation kit of mouse mononuclear cells following manufacturer’s instructions. After PBS rinsing and centrifugation at 800 g for 10 min, the cells were counted using the hemocytometer chamber and the viability of PBMCs was assessed by trypan blue staining. The cells were cultured in DMEM medium supplemented with 10 % fetal calf serum (FCS), 1 % glutamine and 1 % PSA (penicillin + streptomycin) in a 5 % CO_2_ humidity incubator at 37 °C.

**Transwell assay**
Migration assays were performed using transwell chamber. Briefly, 2 × 10^4^ RAW 264.7 or PBMCs cells were suspended in 200 μL serum-free medium and then seeded into the upper chamber. The lower chamber was filled with 700 μL medium containing 10 % FBS. After 48 h, the RAW 264.7 cells on the upper transwell chamber were removed, and the cells on the lower transwell chamber were fixed with 4 % paraformaldehyde, then stained with 0.5 % crystal violet. For PBMCs, the cells in the upper and lower transwell chamber were collected and counted. Five random visual fields were photographed for statistical analysis.

**Cell viability**

Cell viability was measured using Cell Counting Kit-8 (CCK-8) assay. Briefly, B16-F10 cells were seeded at a density of 5 × 10^3^ per well in 96-well plates and incubated with medium spiked with corresponding nanoparticles (40.0 μg/mL) or PBS control for 24 h. The supernatants were then discarded and 100 μL fresh medium containing 10 μL of WST-8 reagent was added. After incubation for another 1 h at 37 °C, absorbance was detected with a spectrophotometer (BioTek, VT, USA) at 450 nm for viability calculation. Each condition was measured with six replicates. In determining the effect of nanoparticles on the activity of RAW 264.7 cells, the concentrations of all nanoparticles were halved compared with those used in B16-F10 cells.

**PI staining**

The treated cells were washed twice with PBS. Then add 10 μL PI (50 μg/mL) to 500 μL cell culture medium, followed by gently mixing and 15-min incubation at 37 °C in the dark. Finally, the cells were visualized under the confocal laser scanning microscopy (CLSM) (Zeiss LSM 880).

**ELISA.**

IL-1α, IL-6, TNF-α and IFN-γ were measured in cell supernatants and mice serum using sandwich ELISA following the manufacturer’s instructions (QuantiCyto® Mouse IL-1α ELISA kit, QuantiCyto® Mouse IL-6 ELISA kit, QuantiCyto® Mouse TNF-α ELISA kit, QuantiCyto® Mouse IFN-γ ELISA kit). The IL-1α, IL-6, IFN-γ and TNF-α recombinant proteins were used as the standard. The standard curve of each analyte covered a range from 10 ng/mL to 500 ng/mL. The preparation of the ELISA kits and the assays were performed according to the ELISA Development Guide. All assays were performed in triplicate. Optical density (OD) is measured at a wavelength of 450 nm with a spectrophotometer.

**Quantitative real time PCR (qRT-PCR)**

Total RNA was extracted using RNAiso plus according to the manufacturer’s protocol and cDNA was synthesized using Reverse Transcriptase M-MLV. Real-time PCR was performed using the BIORAD CFX96 Touch Real-time PCR System with iTaq™ universal SYBR® Green (Bio-Rad, USA) according to the manufacturer’s instructions. The primers used in this study are listed in Supplementary Methods. The expression levels of genes were quantified using the comparative CT method. The expression level of each mRNA was normalized to the level of GAPDH mRNA. The primer sequences: IL-1α (F: AGTCAACTCATTGGCGCTTG, R: GAGAGAGATGGTCAATGGCAGA), IL-1β (F: TGCCACCTTTTGACAGTGATG, R: AAGGTCCACGGGAAAGACAC), IL-6 (F: CAACCACGGCCTTCCCTACT, R: TTCTCATTTCCACGATTTCCCA), TNF-α (F: TGATCGGTCCCCAAAGGGAT, R: TGTCTTTGAGATCCATGCCGT), IFN-β (F: TGATCGGTCCCCAAAGGGAT, R: GCCCTGTAGGTGAGGTTGAT), IFN-γ (F: AGCTCCAAGAAAGGACGAACA, R: GCCCTGTAGGTGAGGTTGAT), NF-kB (F: ATGGCAGACGATGATCCCTAC, R: CGGAATCGAAATCCCCTCTGTT), CCL-2 (F: TAAAAACCTGGATCGGAACCAAA, R: GCATTAGCTTCAGATTTACGGGT), CCL-7 (F: CCACATGCTGCTATGTCAAGA, R: ACACCGACTACTGGTGATCCT) are shown in the Supplementary Methods.

**Western blot**

The cultured cells were lysed for 40 min in RIPA buffer and boiled for 5 min. Total proteins were separated on SDS-PAGE gels and then transferred to PVDF membranes. After blocking with 5 % bovine serum albumin (BSA) for 1 h, the membranes were incubated with respective primary antibodies against PCNA, BAX, c-Caspase3, c-PARP, GAPDH for overnight at 4 °C. Then, the membranes were incubated with the horseradish peroxidase (HRP)-conjugated secondary antibodies, and the signals were detected by the enhanced ECL system with the Western Blot exposure (Bio-Rad, USA).

**Animal models**

C57BL/6 mice (Male, 8 weeks old) were purchased from Beijing HFK Bio-Technology (Beijing, China). The studies were approved by the Committee on Ethics of Animal Experiments of Tongji Hospital, Tongji Medical College, Huazhong University of Science and Technology. In order to introduce the subcutaneous tumor, 5 × 10^6^ B16-F10 cells were resuspended in 100 μL of serum free DMEM medium and subcutaneously injected into the right back of each mouse. We then measured the sizes of subcutaneous tumors every 2 days. The tumor volume was calculated following the formula: volume = (length × width^2^) ×0.5. When the volumes of tumors reached around 100 mm^3^, tumor-bearing mice were randomly divided into five groups. Mice were intravenously injected with corresponding NPs or PBS. The injection volume for each mouse was 100 μL. NPs were dispensed in water with the final concentration of 5.0 mg/mL. After 24 h of injection, the tumor areas of M@A (Light +) and M@AP (Light +) groups were exposed to white light (200 mW cm^-2^) for 20 min. The treatment was repeated every two days for 15 days. Tumor sizes were measured every 2 days.

In another tumor model, 5 ×10^6^ B16-F10 cells were subcutaneously injected into both right and left back of C57BL/6 mice and grew for 7 days. Then, only the right side tumors were treated with corresponding NPs or PBS through intratumoral injection. The injection volume for each mouse was 100 μL. NPs were dispensed in water with the final concentration at 5.0 mg/mL. After 24 h, tumors of right back in M@A (Light +) and M@AP (Light +) groups were exposed to white light (200 mW cm^-2^) for 20 min. Tumor sizes on both right and left back were measured every 2 days.

To construct the lung metastasis model, 5 ×10^6^ EGFP-B16-F10 cells were first subcutaneously injected into the right back of C57BL/6 mice. Meanwhile, additional 5 ×10^6^ EGFP-B16-F10 cells suspended in PBS were intravenously injected into these mice. When volumes of tumors reached around 100 mm^3^, the NPs or PBS were injected via tail vein as described above. After half a month of treatment, lung metastases were evaluated using an *in vivo* imaging system (IVIS^®^) and H&E staining.

**H&E staining**

The tissues were fixed in 4 % paraformaldehyde for 24 h, transferred into 70 % ethanol, and then paraffin embedded. Sections of four-micrometer thick of embedded tissue were deparaffinized andrehydrated, followed by staining with hematoxylin and eosin.

**Pharmacokinetics and bio-distribution of NPs**

To detect the pharmacokinetics of AP and M@AP NPs in vivo, three 8-week-old male C57BL/6 mice were injected with AP or M@AP NPs through the tail veins. Approximate 50 μL blood of each mouse was taken through the posterior orbital vein right before the injection as the blank control. Also, same volume of blood sample was collected at the indicated timepoints post injection. Fluorescence signals of all the blood samples were detected by the spectrofluorometer. To detect the bio-distribution of NPs, AP or M@AP NPs were intravenously injected into the B16-F10 tumor-bearing mice. After 24 h, the mice were dissected, and the distribution of NPs was visualized by using IVIS^®^.

**Hemolysis rate detection**

To detect the hemolysis rate of the NPs, blood from mouse was collected in tubes containing sodium citrate. The RBCs were isolated by centrifugation (1000 rpm, 5 min) and washed several times with PBS. Then, 0.5 mL of the RBCs suspension was mixed with 0.5 mL suspension containing PBS (negative control), M@AP, PLGA, A, P, M, and water (positive control) respectively. The mixture was incubated at room temperature for 2 h and then centrifuged at 5000 rpm for 5 min. The percentage of hemolysis was calculated as follows: Hemolysis% = (sample absorbance – negative control absorbance) / (positive control absorbance – negative control absorbance) × 100%.

**Flow cytometry**

To assess the NP-induced anti-tumor immune effects, the peripheral blood of each group of mice were collected into ETDA-coated tubes and treated twice with ACK (Ammonium-Chloride-Potassium) lysing buffer followed by a 1 mL PBS wash. Then, cells were labeled with anti-CD3, CD4, CD8, CD19, and CD49b antibodies for 20 min and washed twice with PBS. The cells were resuspended in 100 μL FACS buffer and then analyzed by flow cytometry (Beckman, cytoflex S). The gating and cell identification strategy for our basic staining protocol was as follows: after cell doublets and clumps were eliminated by FSC-H vs. FSC-A gating and debris was eliminated using FSC-A vs. SSC-A (R1). Total live cells (R2) were then identified based on the exclusion of a Live/Dead dye. Next, according to the expression of cell marker and negative control, the cells were divided into R3. Total T lymphocyte were identified based on expression of CD3; T lymphocyte subsets were distinguished based on their expression of CD4 and CD8; The identity of B lymphocyte and natural killer (NK) cells was confirmed by their expression of CD19, and CD49b, respectively.

**Immunofluorescence staining**

The anti-tumor effects of NPs were evaluated by immunofluorescence staining. Briefly, tumors from animal model of each group were fixed with formalin and embedded with paraffin. The 4 μm plate sections were then baked at 65 °C for 2 h, deparaffinized in xylene, rehydrated through a graded series of ethanol (100, 95, 85, and 75 %), and washed with PBS. The sections were microwaved in citrate buffer (pH =6.0) for 15 min for antigen retrieval. Next, the sections were incubated in 3 % BSA at 37 °C for 30 min, followed by incubation of primary antibodies overnight at 4 °C. The antibodies against c-Caspase3, Ki67, CD4, CD8, F4/80, CD11b and CD86 were used in this experiment. After washing thrice with PBS, the sections were subsequently incubated with secondary antibodies followed by Hoechst staining for 15 min at room temperature in the dark. Finally, the cells were visualized under the CLSM.

**TUNEL Staining**

According to the manufacturer's protocol, TUNEL staining was conducted to detect apoptotic cells using a commercial kit (TUNEL Bright Green Apoptosis Detection Kit, Vazyme, China).

**Statistical analysis**

All data are expressed as mean ± SD. Statistical comparisons were performed using Chi-square test, unpaired Student’s t tests, one-way ANOVA or two-way ANOVA. Statistical analysis was performed by SPSS (version 13.0) and GraphPad Prism (version 8.1.2; GraphPad Software). The significance level was set at *p < 0.05*.

**Reference**

1 Tan, CF, Azmansah, SAB and Zhu H et al. Spontaneous Electroless Galvanic Cell Deposition of 3D Hierarchical and Interlaced S-M-S Heterostructures. *Advanced Materials* 2017;29:Artn 1604417.

2 Chen Z, Zhao PF and Luo ZY et al. Cancer Cell Membrane-Biomimetic Nanoparticles for Homologous-Targeting Dual-Modal Imaging and Photothermal Therapy. *Acs Nano* 2016;10:10049-57.

3 Zhu JY, Zheng DW and Zhang MK et al. Preferential Cancer Cell Self-Recognition and Tumor Self-Targeting by Coating Nanoparticles with Homotypic Cancer Cell Membranes. *Nano Lett* 2016;16:5895-901.


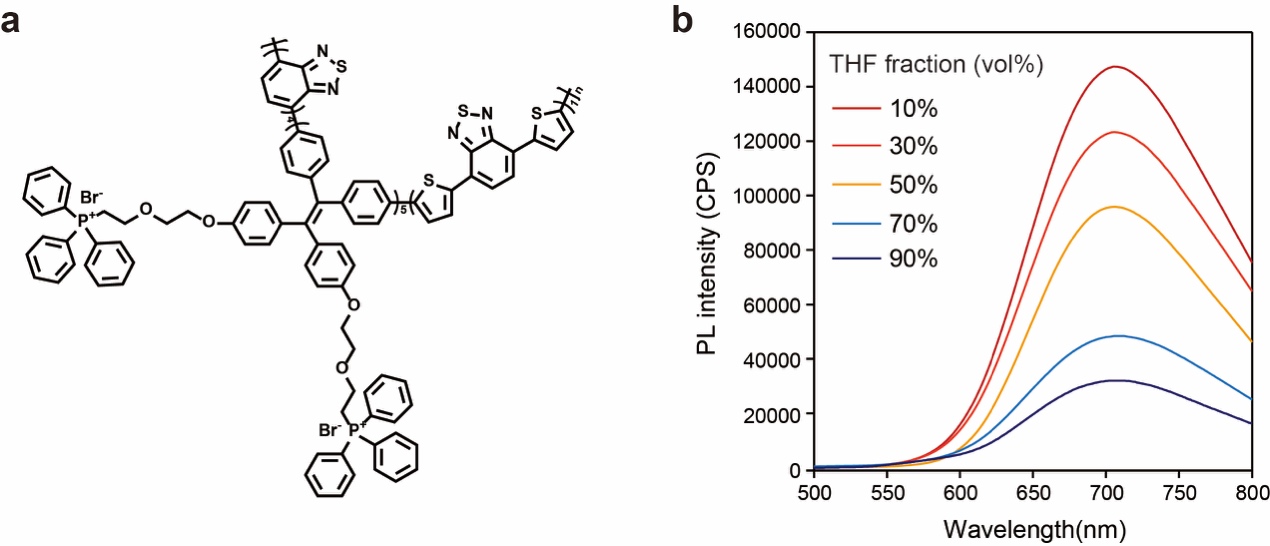


**Figure s1**. (a) The structure of P2-PPh3. (b) PL spectra of P2-PPh_3_ in DMSO/THF (*v/v*) mixture. Dye concentration: 10 μM; excitation wavelength: 506 nm.


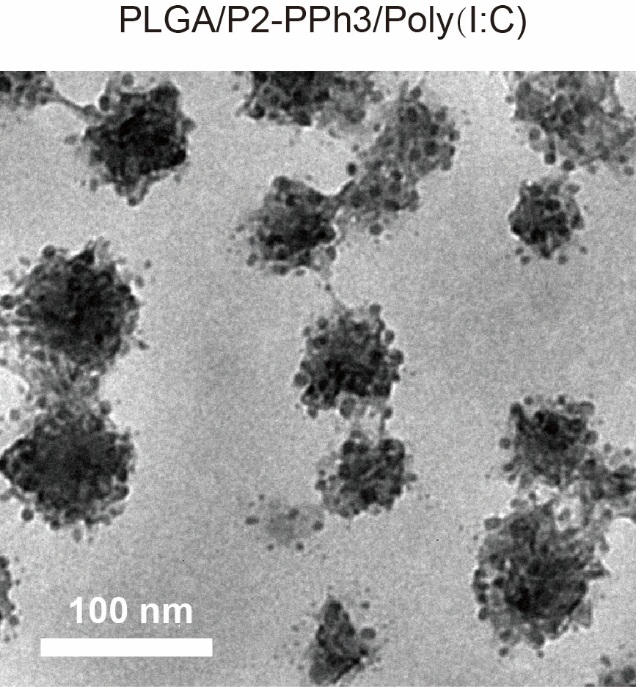


**Figure s2**. Representative transmission electron microscopy (TEM) image of PLGA/P2-PPh3/ Poly(I:C). Scale bar: 100 nm.


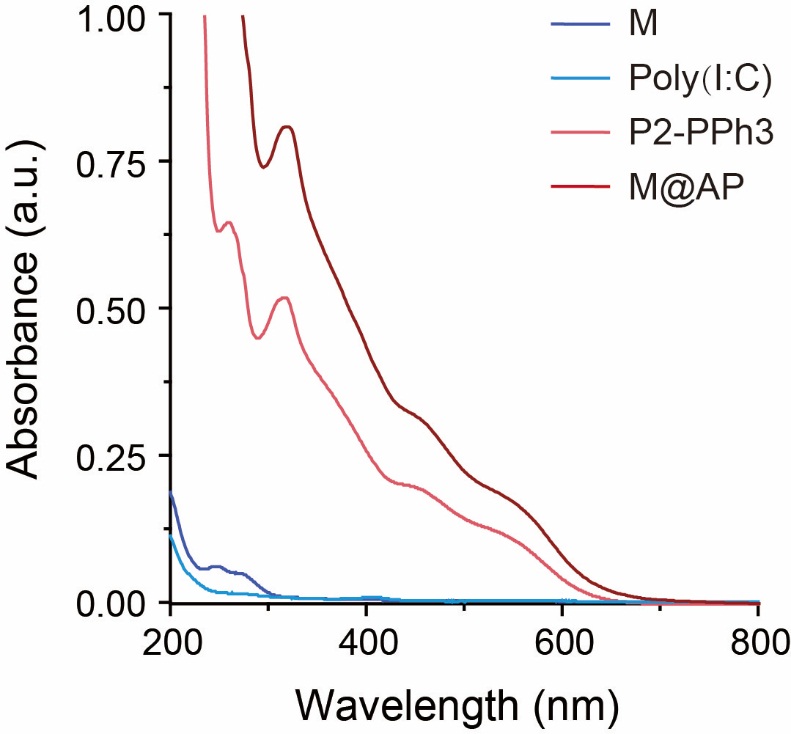


**Figure s3**. UV spectra of M (RBCs membrane), Poly(I:C), P2-PPh3 and M@AP.


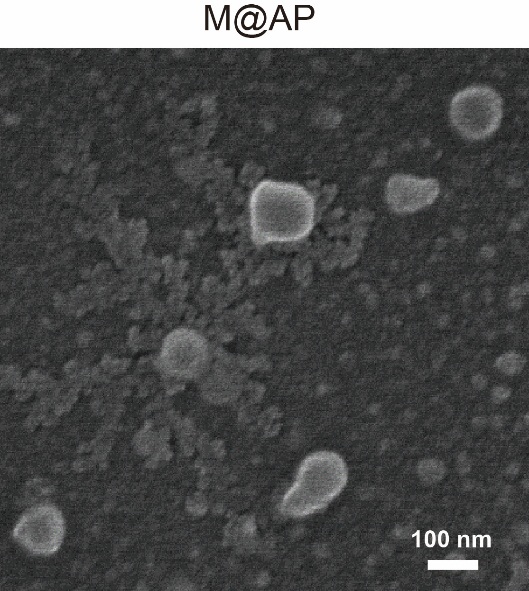


**Figure s4**. Representative scanning electron microscopy (SEM) image of M@AP. Scale bar: 100 nm.


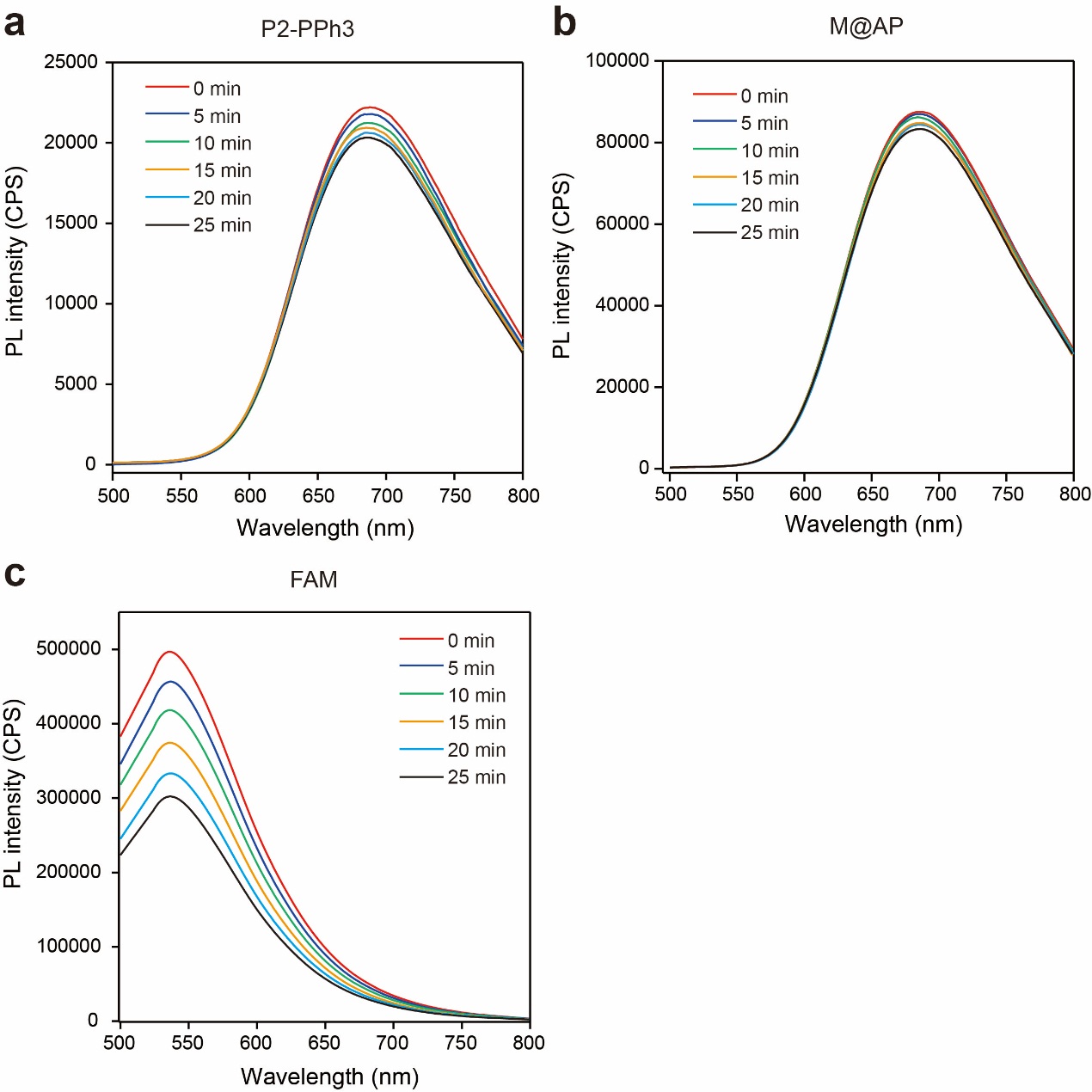


**Figure s5**. Response of (a) P2-PPh3, (b) M@AP and (c) 5-Carboxyfluorescein (FAM) fluorescence to photobleaching. White light intensity: 100 mW cm^-2^. Dye concentration: P2-PPh3 10 μM, M@AP 1.0 mg/mL, FAM 10 μM; excitation wavelength: P2-PPh3 506 nm, M@AP 506 nm, FAM 488 nm.


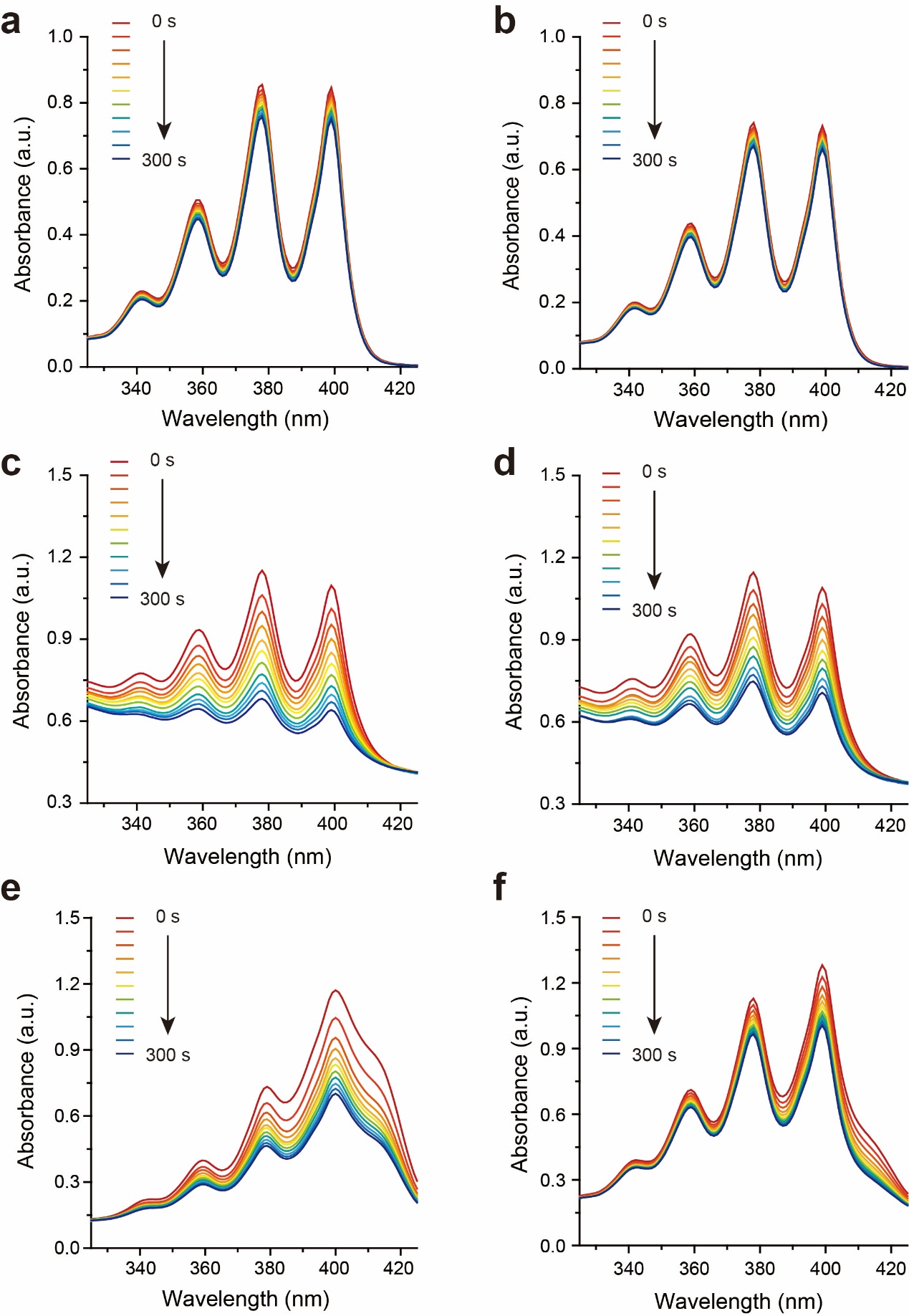


**Figure s6**. The ability of (a) water, (b) M@P, (c) M@AP, (d) M@AP (after photobleaching), (e) Ce6 and (f) Ce6 (after photobleaching) to produce ROS. White light intensity: 20 mW cm^-2^.


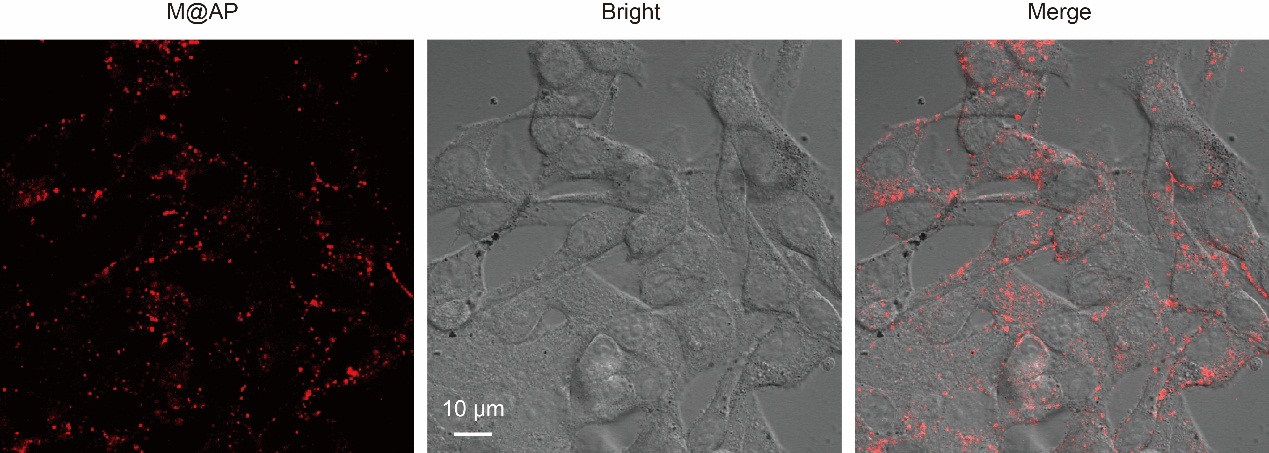


**Figure s7**. B16-F10 cells were incubated with M@AP for 8 h, and then the fluorescence signals were detected by confocal laser scanning microscopy (CLSM). P2-PPh3: Excitation 488 nm, Emission 660-700 nm. Scale bar: 10 μm.


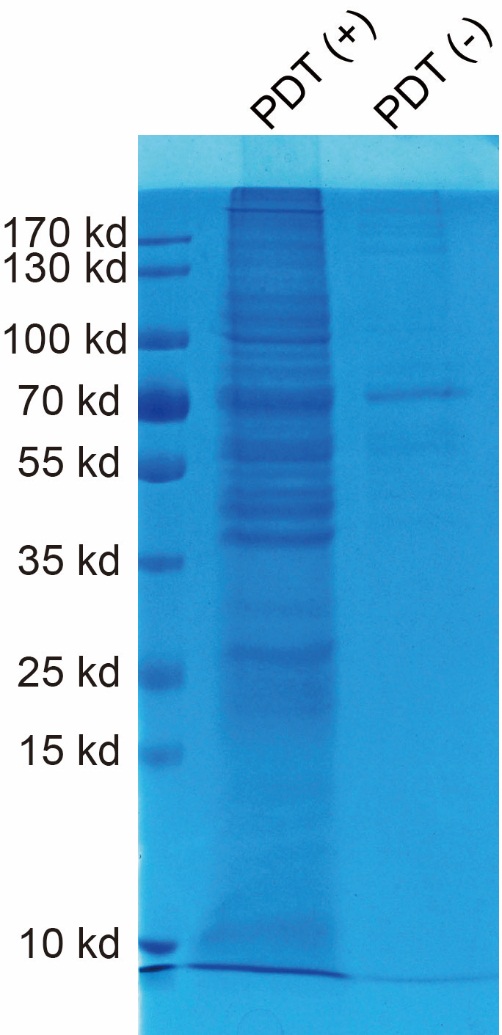


**Figure s8**. SDS-PAGE pattern of proteins in the culture medium of PDT-treated B16-F10 cells.


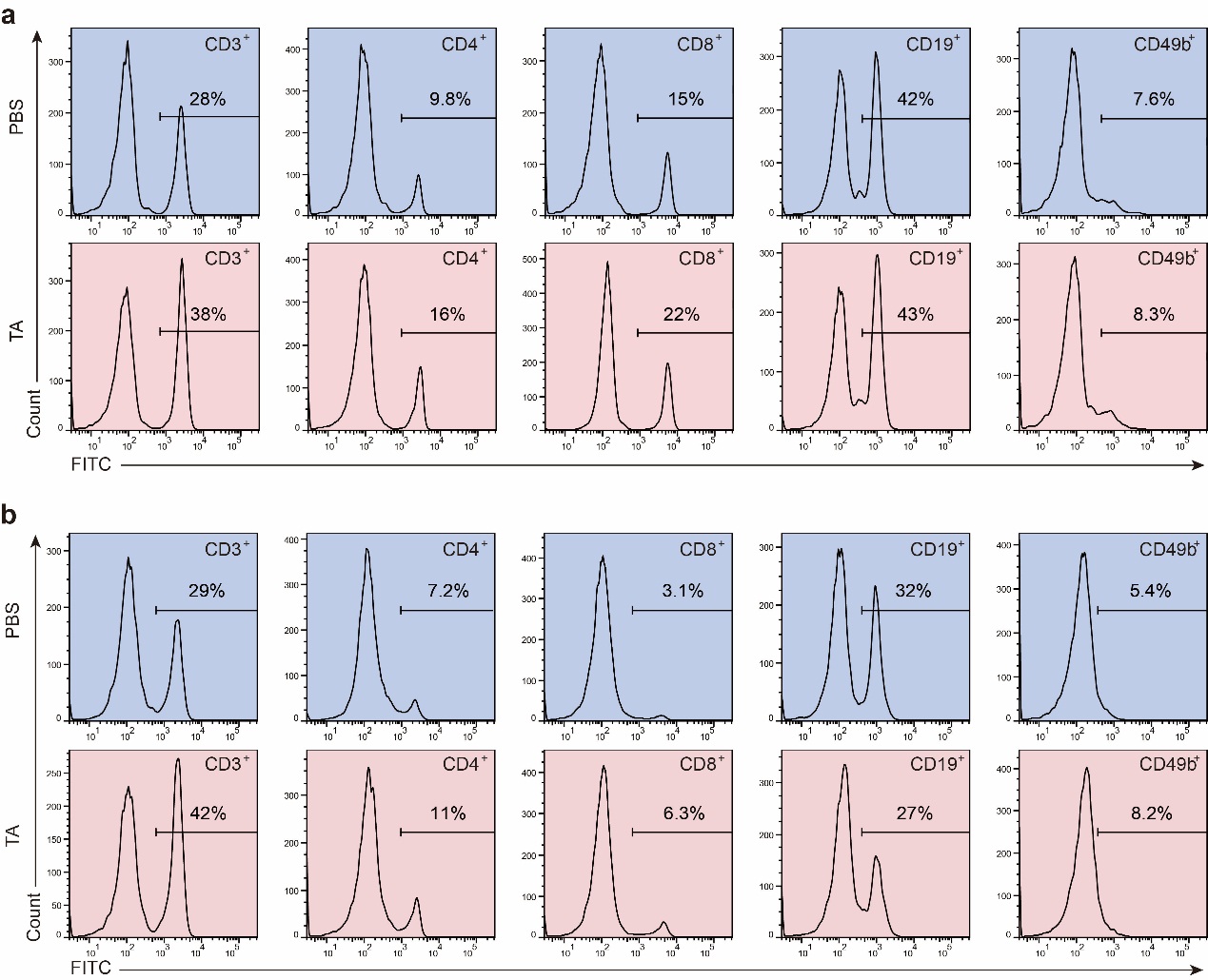


**Figure s9.** The proportion of CD3^+^, CD4^+^, CD8^+^, CD19^+^ or CD49^+^ cells in (a) peripheral blood and (b) spleen were detected by flow cytometry.


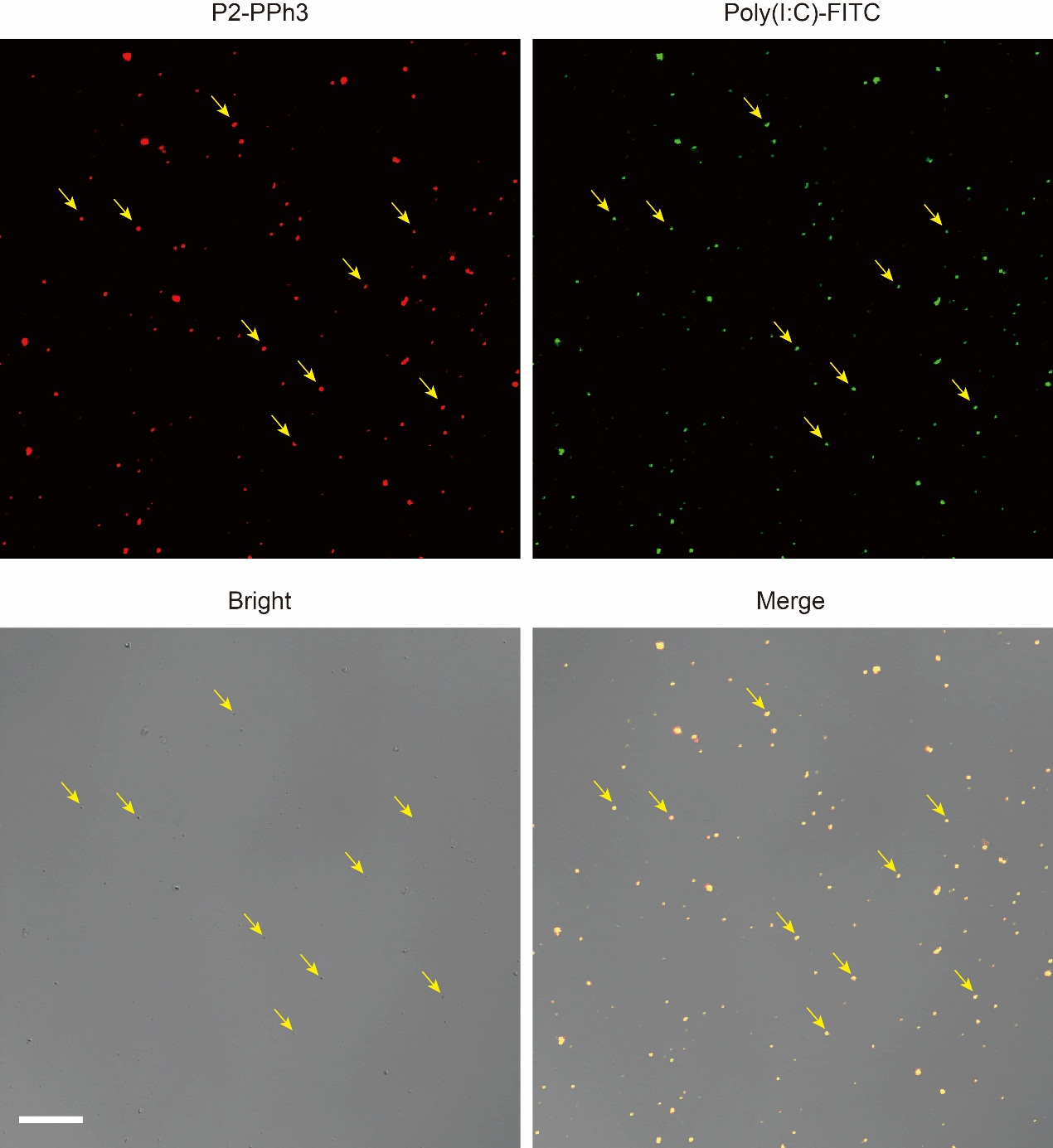


**Figure s10**. The fluorescence and morphology of M@AP-FITC were observed by CLSM. The yellow arrow indicates M@AP-FITC. Scale bar =10 μm.


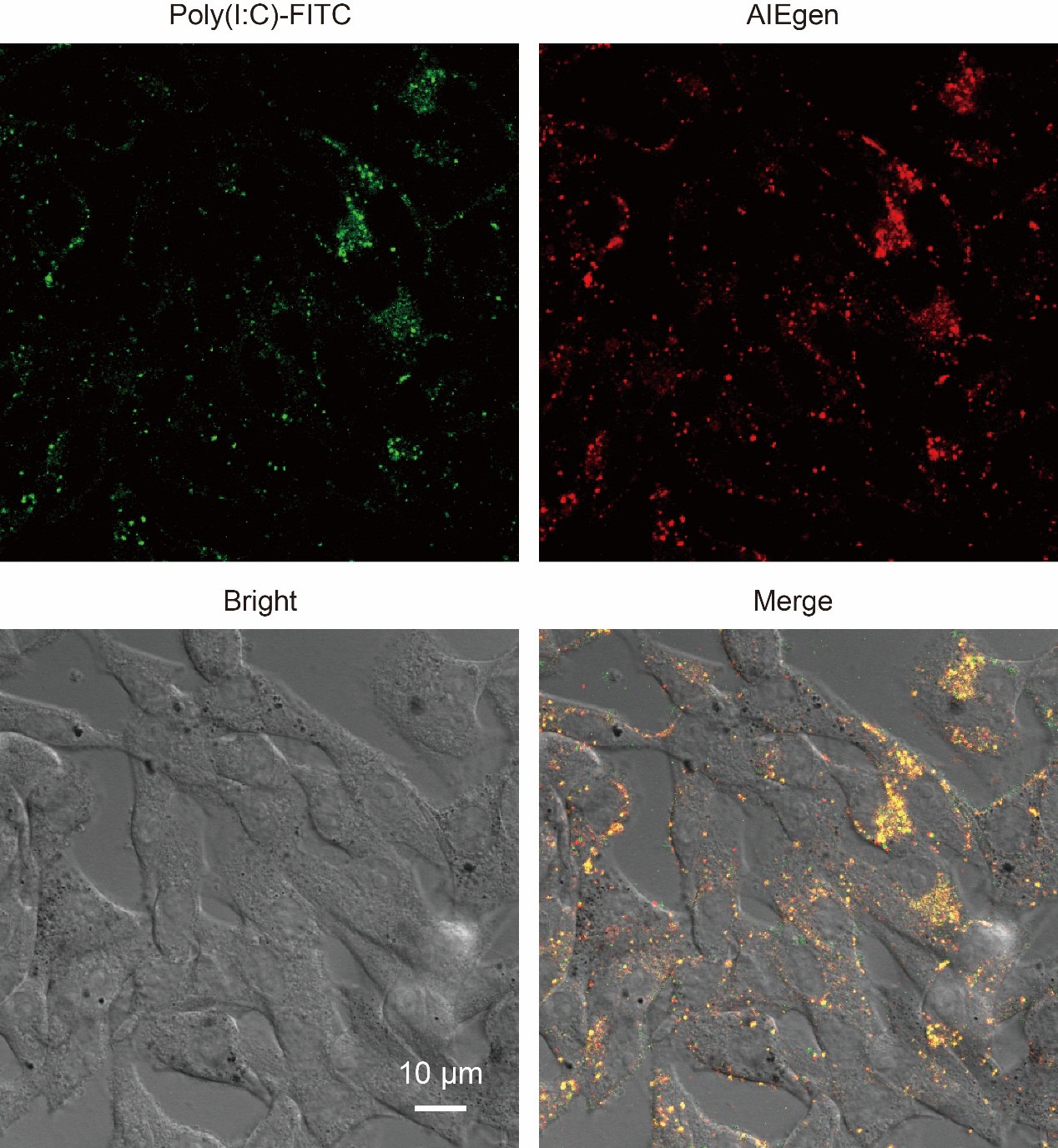


**Figure s11**. The B16-F10 cells were incubated with M@AP-FITC for 8 h, and then the fluorescence signals were detected by CLSM. FITC: Excitation 488 nm, Emission 500-540 nm. P2-PPh3: Excitation 488 nm, Emission 660-700 nm. Scale bar: 10 μm.


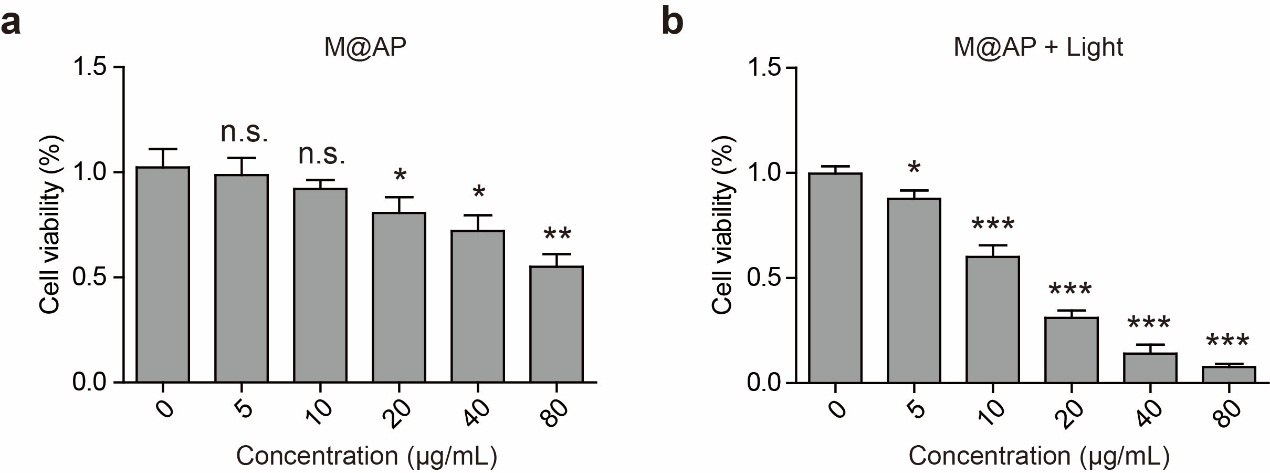


**Figure s12**. (a) The effect of M@AP on the viability of B16-F10 cells in the absence of light irradiation. (b) The effect of M@AP on the viability of B16-F10 cells under light irradiation (Light intensity: 100 mW cm^-2^; Irradiation time: 3 min). The data were reported as mean ± SD and analyzed by two-sided Student’s t-test. * *p*< 0.05，** *p*< 0.01, *** *p*< 0.001. *n.s*. not significant.


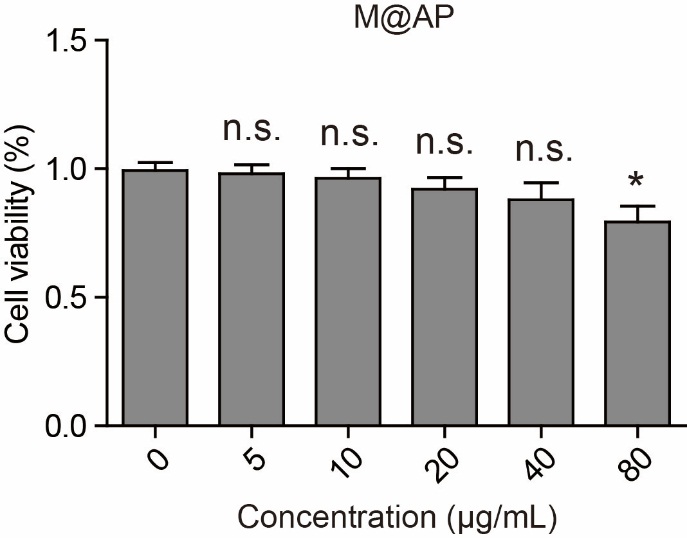


**Figure s13**. The effect of M@AP on the viability of RAW 264.7 cells in the absence of light irradiation. The data were reported as mean ± SD and analyzed by two-sided Student’s t-test. * *p*< 0.05, *n.s*. not significant.


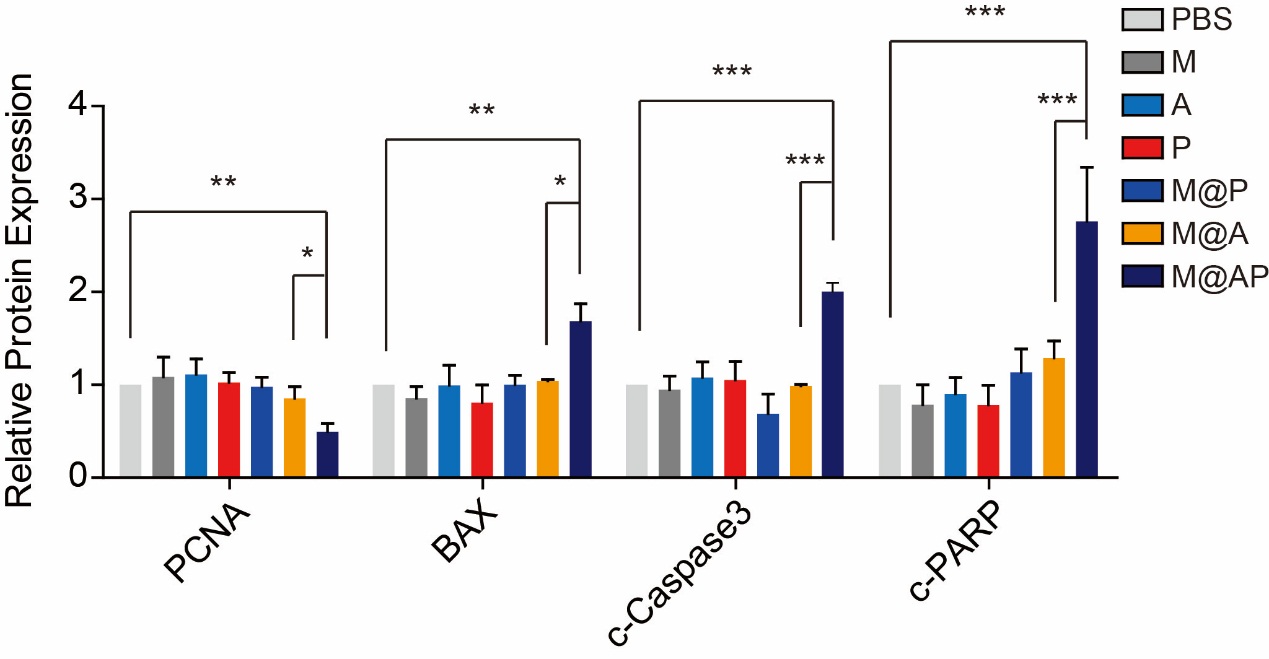


**Figure s14**. The expression levels of PCNA, BAX, c-Caspase3 and c-PARP in B16-F10 cells treated with different biomaterials (PBS, M, A, P, M@P, M@A, and M@AP) were detected by Western blot. * *p*< 0.01, ** *p*< 0.01, *** *p*< 0.001.


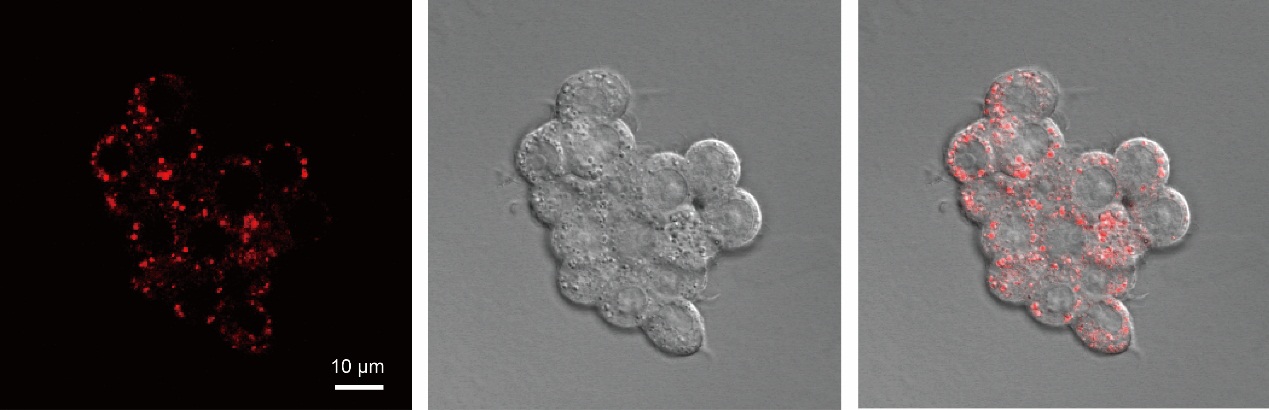


**Figure s15.** The RAW 264.7 cells were incubated with M@AP for 8 h, and then the fluorescence signals were detected by CLSM. P2-PPh3: Excitation 488 nm, Emission 660-700 nm. Scale bar: 10 μm.


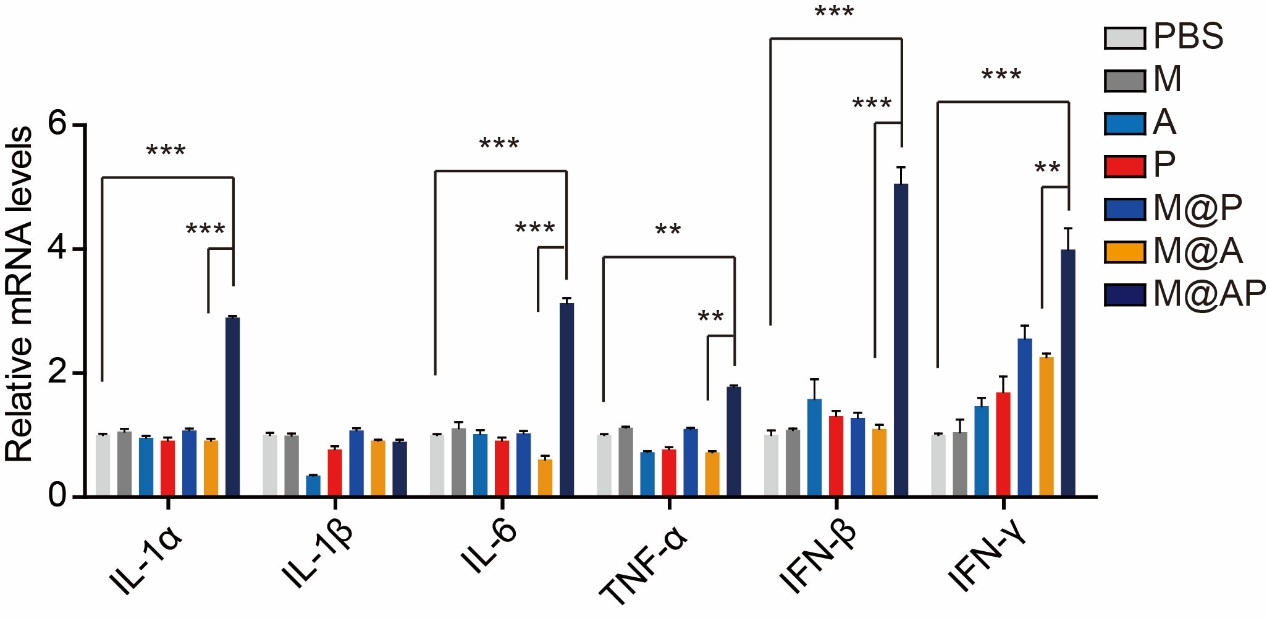


**Figure s16.** The mRNA levels of immune factors in RAW 264.7 cells treated with PBS, M, A, P, M@P, M@A and M@AP were detected by qRT-PCR. ** *p*< 0.01, *** *p*< 0.001.


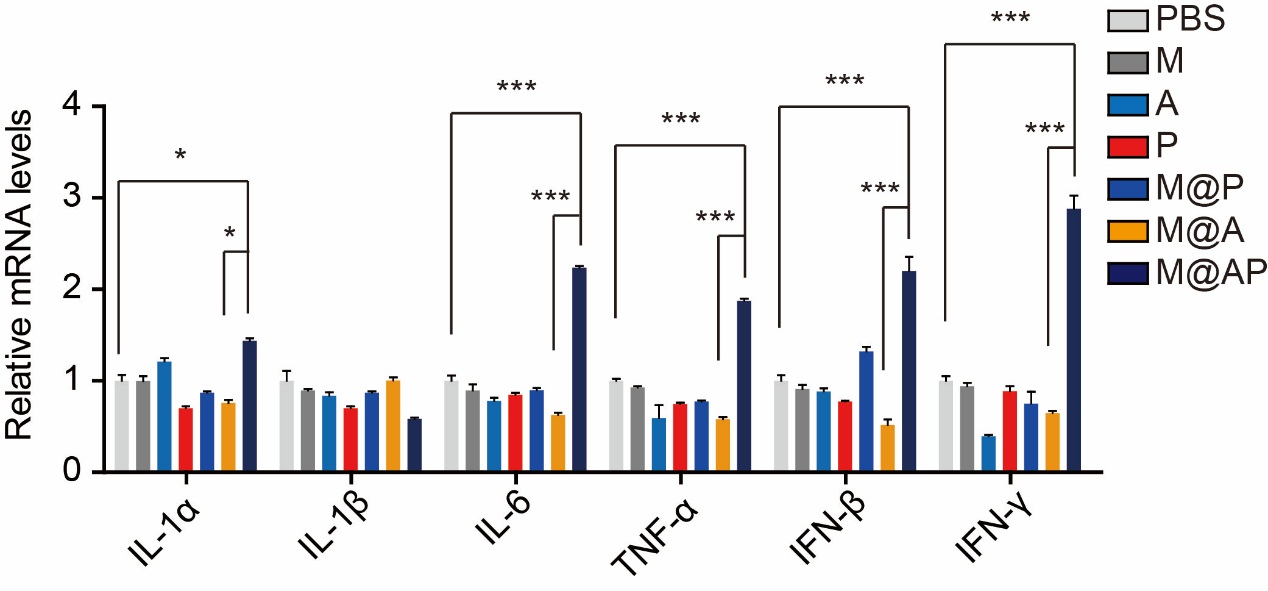


**Figure s17**. The mRNA levels of immune factors in bone marrow-derived macrophages (BMDMs) treated with PBS, M, A, P, M@P, M@A, and M@AP were detected by qRT-PCR. * *p*< 0.01, *** *p*< 0.001.


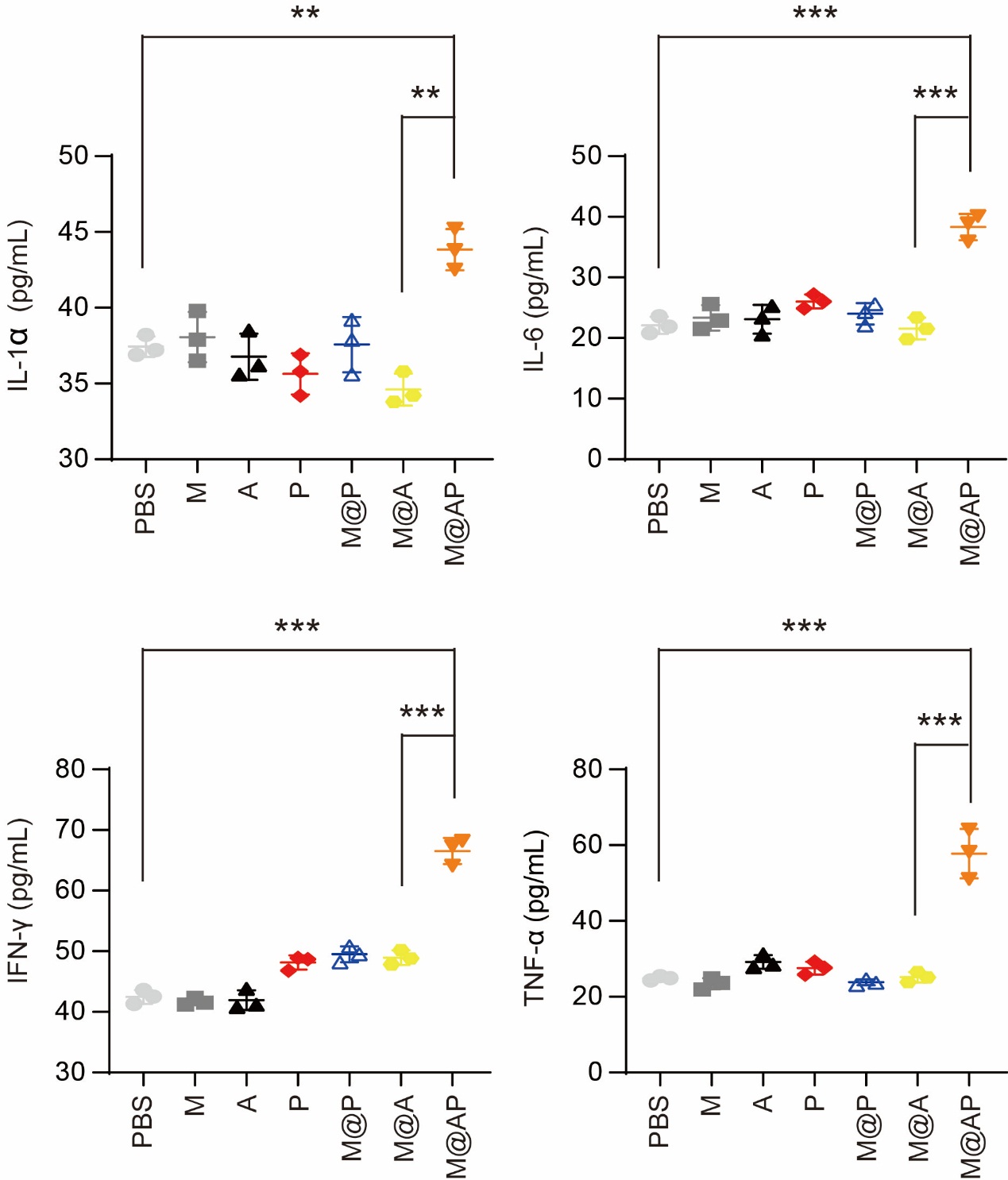


**Figure s18**. The protein levels of immune factors in BMDMs treated with PBS, M, A, P, M@P, M@A, and M@AP were detected by ELISA. ** *p*< 0.01, *** *p*< 0.001.


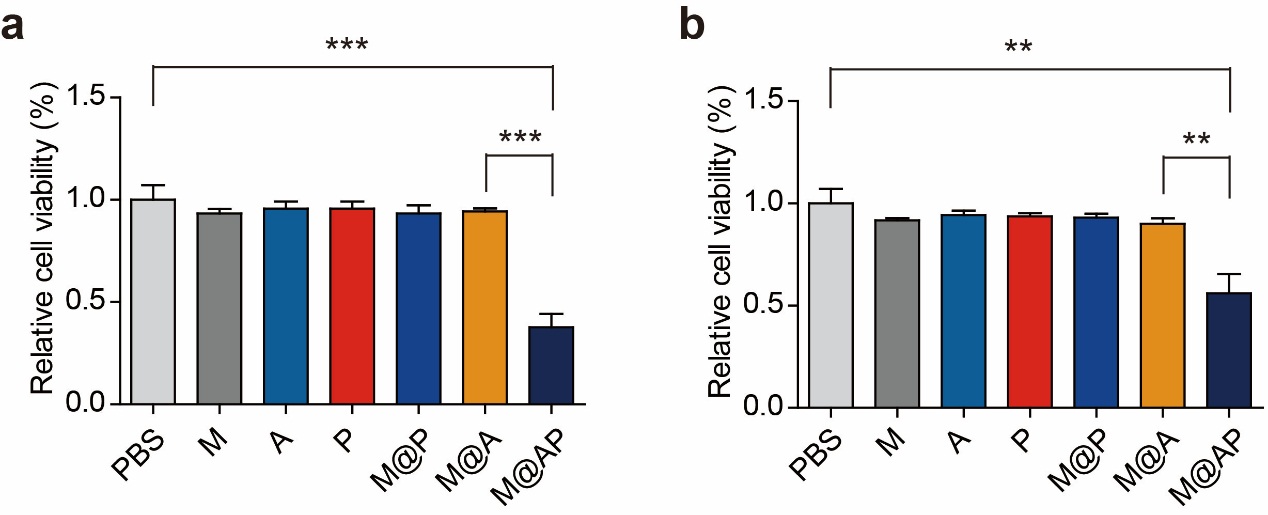


**Figure s19**. (a) EGFP-B16-F10 and (b) PBMCs were pretreated with PBS, M, A, P, M@P, M@A, and M@AP, respectively, and then co-cultured for 18 h. Then the apoptosis of EGFP-B16-F10 cells was detected. The data were reported as mean ± SD and analyzed by two-sided Student’s t-test (n =3). ** *p*< 0.01, *** *p*< 0.001.


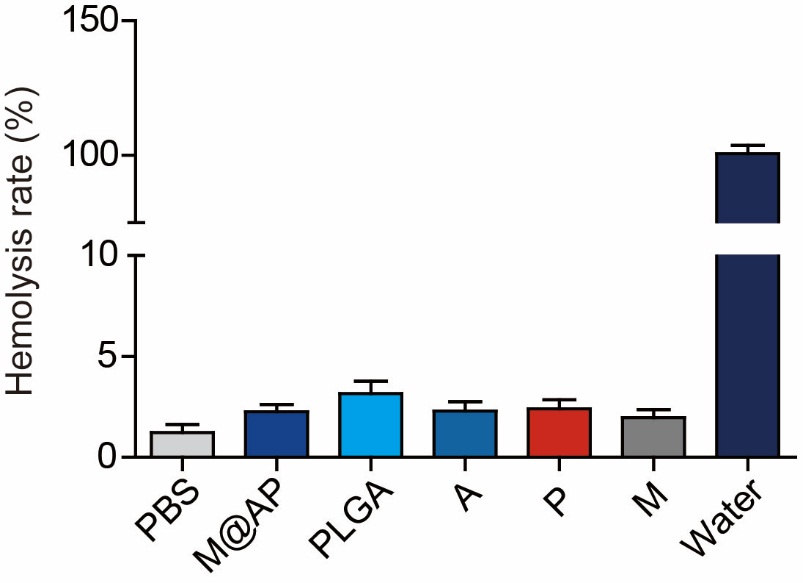


**Figure s20**. Hemolysis test of PBS (negative control), M@AP, PLGA, A, P, M, and water (positive control).


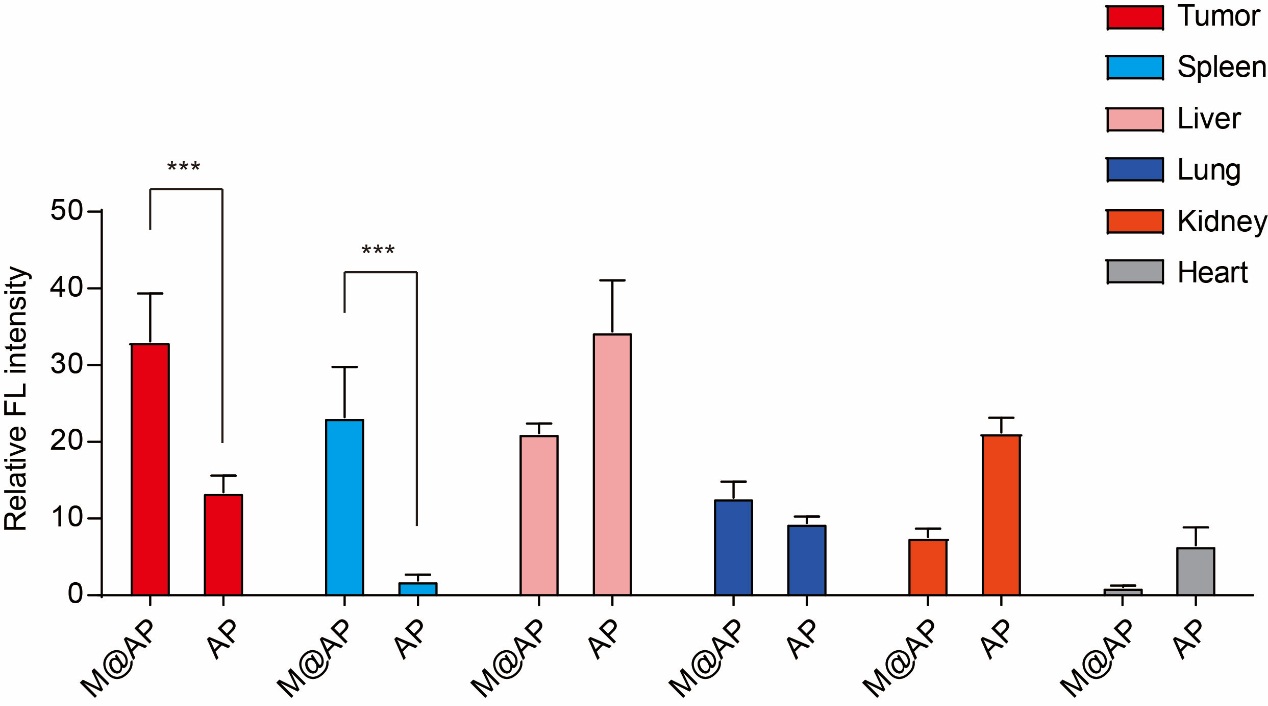


**Figure s21**. The relative FL intensity of AP and M@AP in the tumor, heart, liver, spleen, lungs, and kidneys after injection for 24 h. The data were reported as mean ± SD and analyzed by two-sided Student’s t-test (n =3). *** *p*< 0.001.


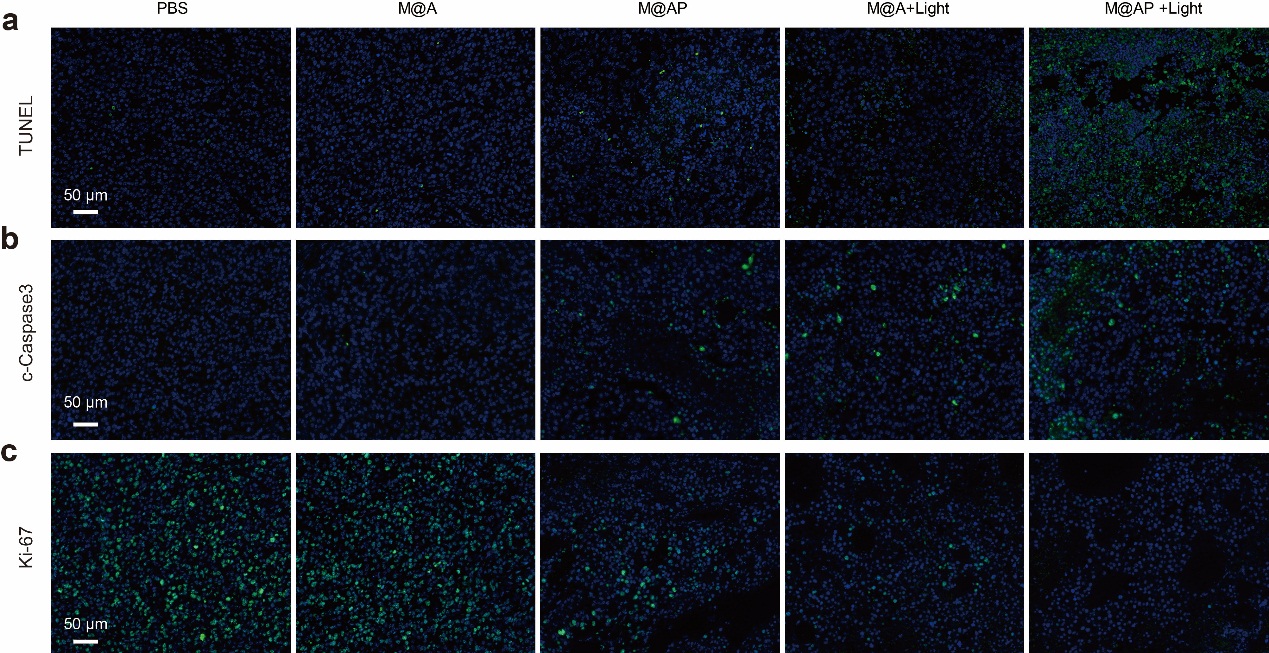


**Figure s22**. TUNEL staining, c-Caspase3 and Ki-67 expression in tumor (full-scale images). (a) Representative TUNEL staining of tumor tissue in unilateral B16-F10 tumor-bearing mice model. The expression levels of (b) c-Caspase3 and (c) Ki-67 in tumor tissues.


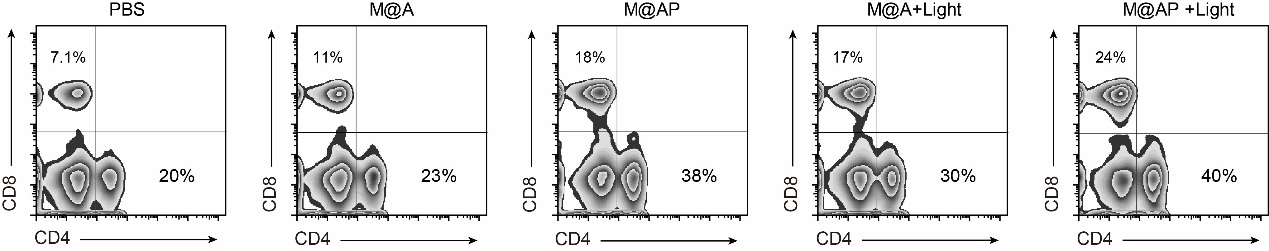


**Figure s23**. The proportion of CD4^+^ and CD8^+^ cells in the peripheral blood of B16-F10 tumor-bearing mice after different treatment.


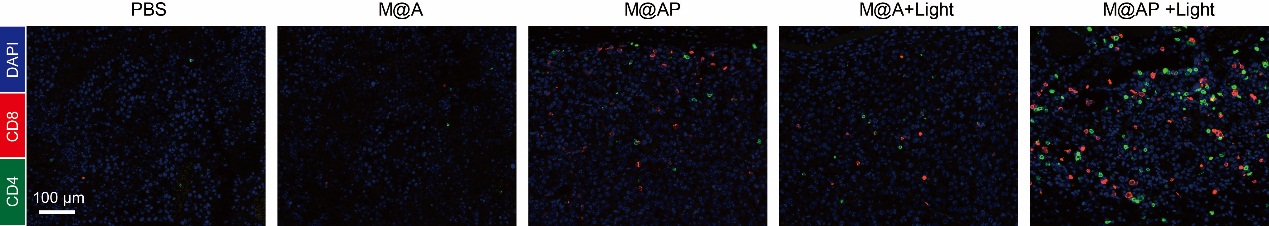


**Figure s24**. CD4^+^ and CD8^+^ T [cells](javascript:;) in tumor tissues (full-scale images).


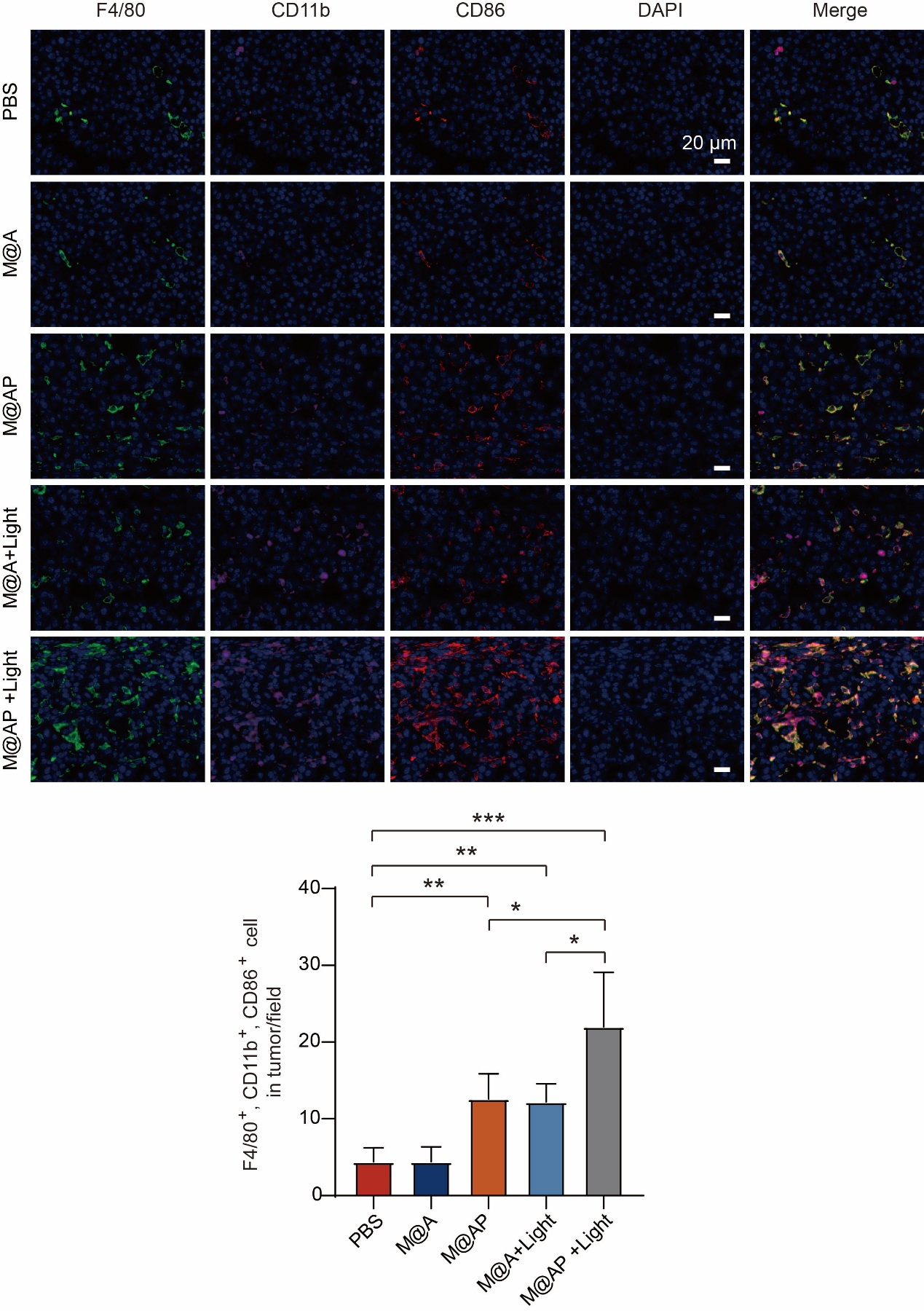


**Figure s25**. The abundance of M1 macrophages (F4/80^+^, CD11b^+^ and CD86^+^) in tumor tissues. Scale bar: 20 μm. The data were reported as mean ± SD and analyzed by two-sided Student’s t-test (n =3). * *p*< 0.05，** *p*< 0.01, *** *p*< 0.001.


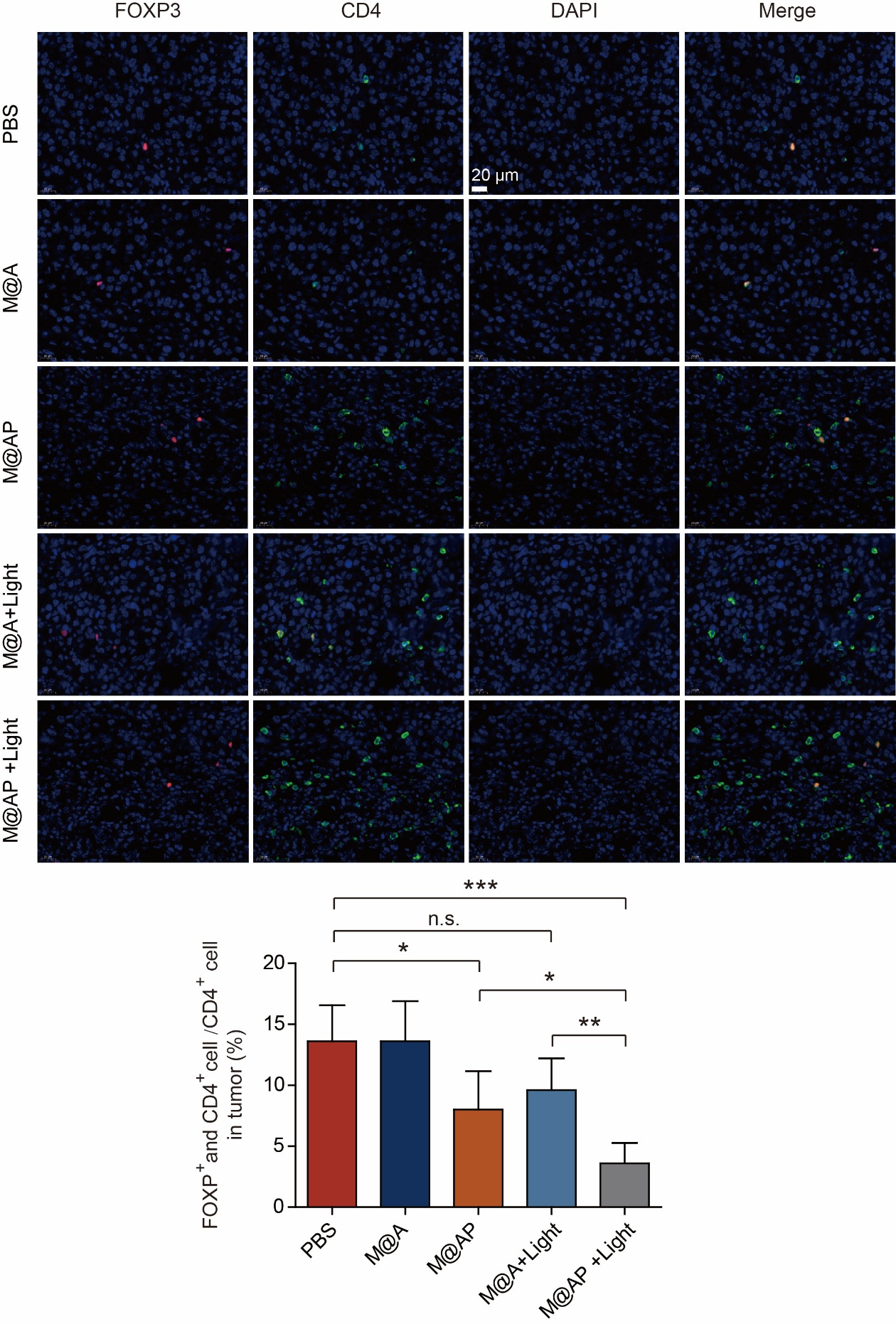


**Figure s26**. Percentage of regulatory T cells (CD4^+^ and FOXP3^+^) in CD4^+^ T cells. Scale bar: 20 μm. The data were reported as mean ± SD and analyzed by two-sided Student’s t-test (n =3). * *p*< 0.05，** *p*< 0.01, *** *p*< 0.001, *n.s*. not significant.


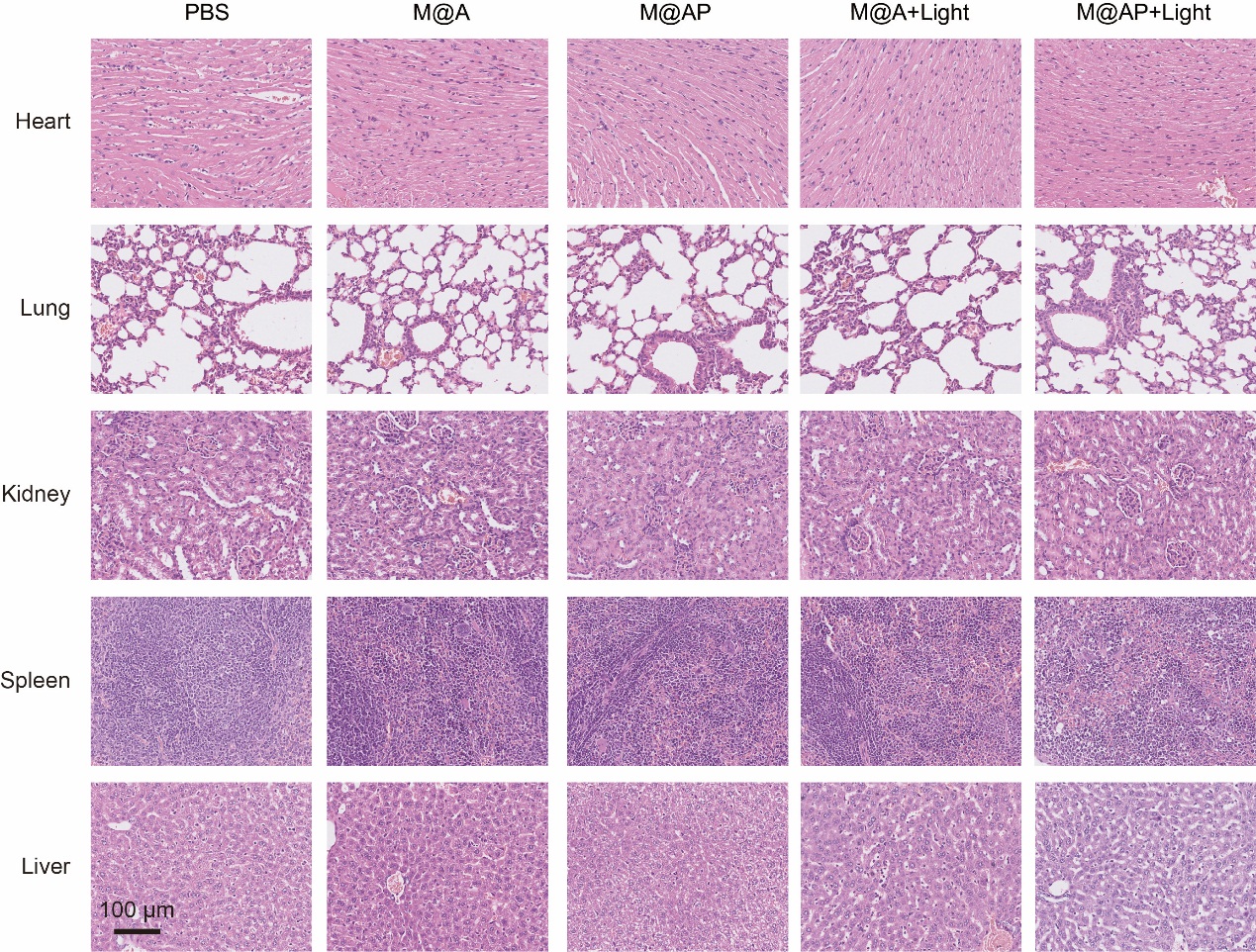


**Figure s27**. Histological examination of the heart, lungs, kidneys, spleen, and liver. Scale bar: 100 μm.


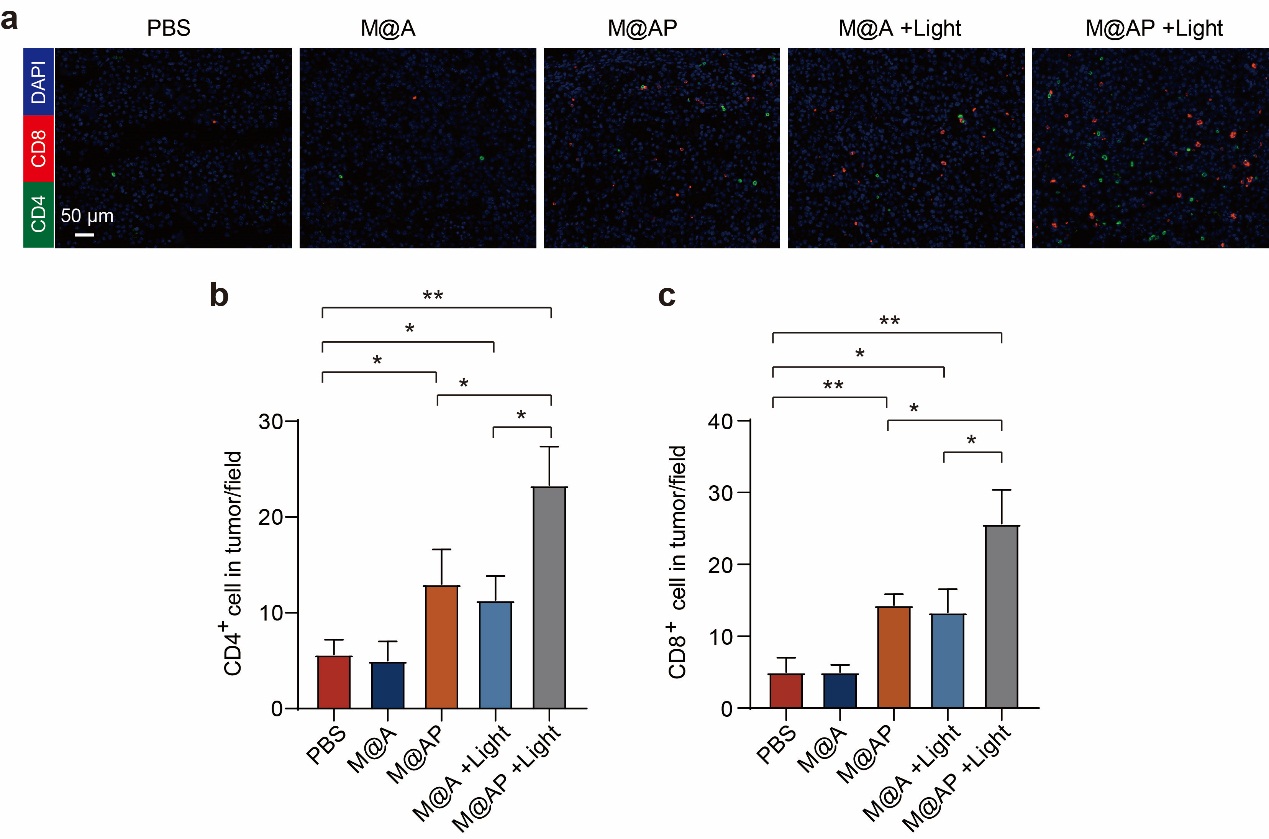


**Figure s28**. (a) The abundance of (b) CD4^+^ and (c) CD8^+^ T cell in tumor tissues (full-scale images). The data were reported as mean ± SD and analyzed by two-sided Student’s t-test (n =3). * *p*< 0.05，** *p*< 0.01.


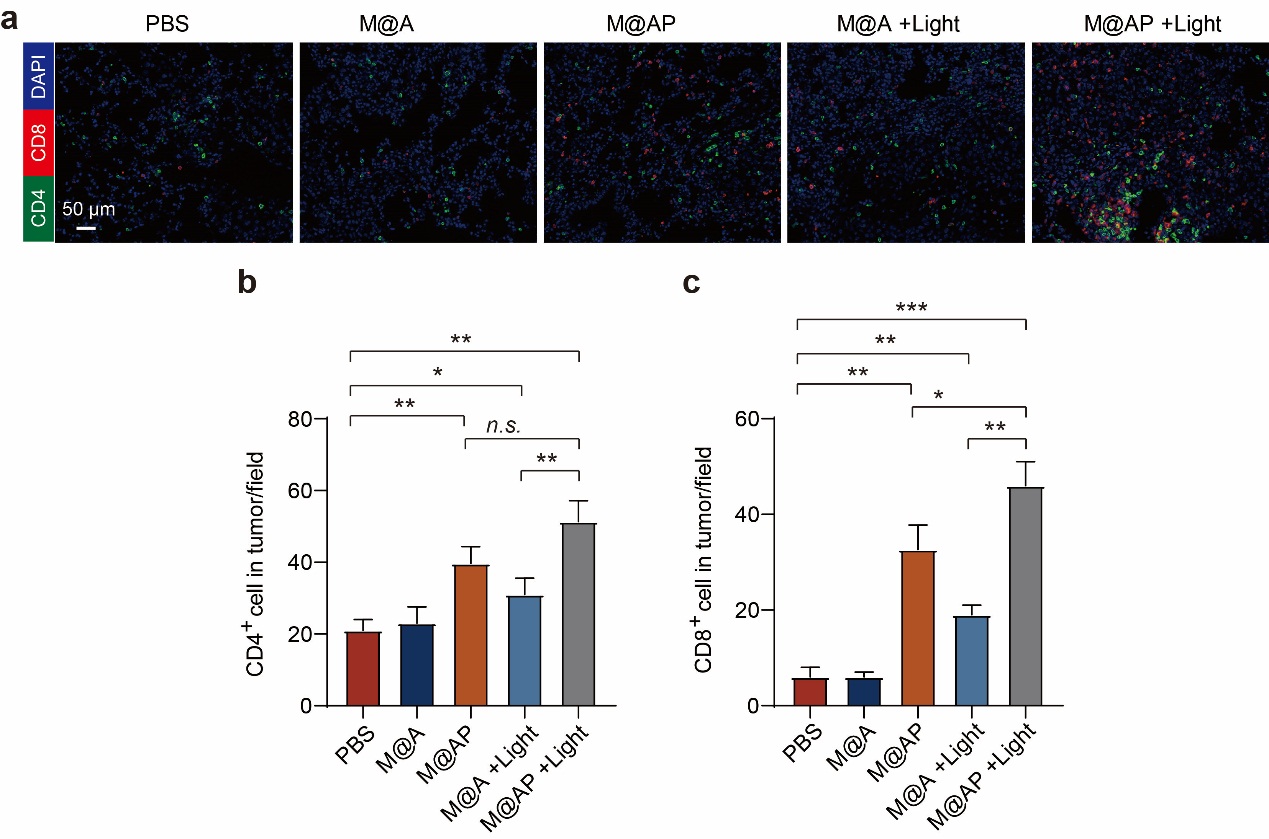


**Figure s29**. (a) The abundance of (b) CD4^+^ and (c) CD8^+^ T cell in lung (full-scale images). The data were reported as mean ± SD and analyzed by two-sided Student’s t-test (n =3). * *p*< 0.05，** *p*< 0.01, *** *p*< 0.001, *n.s*. not significant.
